# Role of Heat-Stable Enterotoxins in the Induction of Early Immune Responses in Piglets after Infection with Enterotoxigenic *Escherichia coli*

**DOI:** 10.1371/journal.pone.0041041

**Published:** 2012-07-17

**Authors:** Michaela Loos, Marisa Geens, Stijn Schauvliege, Frank Gasthuys, Jan van der Meulen, J. Daniel Dubreuil, Bruno M. Goddeeris, Theo Niewold, Eric Cox

**Affiliations:** 1 Laboratory of Veterinary Immunology, Faculty of Veterinary Medicine, Ghent University, Merelbeke, Belgium; 2 Laboratory of Livestock Physiology, Immunology and Genetics, Department of Biosystems, Faculty of Bioscience Engineering, Katholieke Universiteit Leuven, Leuven, Belgium; 3 Department of Surgery and Anaesthesia of Domestic Animals, Faculty of Veterinary Medicine, Ghent University, Merelbeke, Belgium; 4 Department of Animal Nutrition, Wageningen UR Livestock Research, Lelystad, The Netherlands; 5 Département de Pathologie et Microbiologie, Faculté de Médicine Vétérinaire, Université de Montréal, Québec, Canada; Universität Münster, Germany

## Abstract

Enterotoxigenic *Escherichia coli* (ETEC) strains that produce heat-stable (ST) and/or heat - labile (LT) enterotoxins are cause of post – weaning diarrhea in piglets. However, the relative importance of the different enterotoxins in host immune responses against ETEC infection has been poorly defined. In the present study, several isogenic mutant strains of an O149:F4ac^+^, LT^+^ STa^+^ STb^+^ ETEC strain were constructed that lack the expression of LT in combination with one or both types of ST enterotoxins (STa and/or STb). The small intestinal segment perfusion (SISP) technique and microarray analysis were used to study host early immune responses induced by these mutant strains 4 h after infection in comparison to the wild type strain and a PBS control. Simultaneously, net fluid absorption of pig small intestinal mucosa was measured 4 h after infection, allowing us to correlate enterotoxin secretion with gene regulation. Microarray analysis showed on the one hand a non-toxin related general antibacterial response comprising genes such as *PAP, MMP1* and *IL8*. On the other hand, results suggest a dominant role for STb in small intestinal secretion early after post-weaning infection, as well as in the induced innate immune response through differential regulation of immune mediators like interleukin 1 and interleukin 17.

## Introduction

Enterotoxigenic *Escherichia coli* (ETEC) are a diverse group of pathogens that are characterized by the ability to colonize the small intestine while producing enterotoxins, which induce severe secretory diarrhea [1], [2]. ETEC strains are recognized as one of the major causes of dehydrating diarrhea in children in developing countries and as an important causative agent of traveler’s diarrhea [3], [4]. ETEC can also cause diarrhea in newborn calves and in suckling or recently weaned piglets. The apparent similarities between porcine and human ETEC infections [5], [6] and between both species, makes the pig an excellent intestinal model.

Virulent ETEC strains produce fimbriae allowing the bacteria to colonize a host expressing the corresponding fimbrial receptors. ETEC that cause porcine post-weaning diarrhea are frequently of the O149 serotype and carry the F4 (K88) adhesin that permits adhesion of the bacteria to pig intestines [7], [8]. Furthermore, ETEC strains are known to produce heat-labile enterotoxin (LT) and heat-stable enterotoxins a and b (STa, STb), which induce water and electrolyte loss from the intestine [9]. An individual ETEC strain may produce one or more enterotoxins [10], [11], [12], which may explain differences in virulence. However, only limited information is available concerning the contribution of the different enterotoxins to the virulence of a strain. The relative importance of LT as a virulence factor compared to STb has been demonstrated in a gnotobiotic piglet infection model, using isogenic deletion mutants of a naturally occurring porcine pathogen or by complementing a non-pathogenic *E. coli* strain with either STb or LT [13], [14], [15]. Also, LT has well known adjuvant capacities [16] and is able to down-regulate innate host responses *in vitro* [17], [18]. Additionally, studies with the human epithelial cell line HCT-8 suggest a role for STa in the induction of an IL-8 response [19].

Little is known about the induction of host early immune responses after infection with ETEC and how these innate immune responses relate to the resolution of infection [2], [19]. In a recent study, increased fecal IL-8 levels appeared to be important in resolving ETEC infection [20].

In order to investigate the role of the various enterotoxins, in the present study, various mutant strains of the ETEC reference strain GIS26 (O149:F4ac^+^, LT^+^ STa^+^ STb^+^) lacking one or more enterotoxins were generated. The “*in vivo* small intestinal segment perfusion” (SISP) technique [21], [22] was used to correlate pathogen induced gene expression by microarray analysis with a functional response (fluid absorption).

## Materials and Methods

### Animals

Eight 5-week-old female piglets (Belgian Landrace), weaned on day 28, were purchased from a commercial piggery. The animal experiment was reviewed and approved by the Ethical Committee of the Faculty of Veterinary Medicine at Ghent University, in accordance with the Belgian law on animal experimentation (EC2008/077). The presence of the F4 receptor on the brush border of small intestinal enterocytes was confirmed on intestinal villi of each piglet as described by Van den Broeck *et al.* [23].

### Bacterial Strain and Mutants

The hemolytic *E. coli* strain GIS26, serotype O149:K91:F4ac (GIS26 WT), producing the heat-labile enterotoxin (LT^+^) and heat-stable enterotoxin types a and b (STa^+^, STb^+^), was used to generate mutant strains, lacking one or more enterotoxins. Mutants were generated using the bacteriophage lambda recombinase system (λ-Red) as described by Datsenko and Wanner [24]. Briefly, L-arabinose induced GIS26 transformants carrying the Red helper plasmid (pKD46) were grown at 30°C to an OD_600_ of 0.6 and electroporated with PCR products using standard procedures. The PCR products were generated by primers targeting an antibiotic resistance cassette (chloramphenicol or kanamycin) with Flippase recognition target (FRT) sites from a template (pKD3 or pKD4) but flanked by 50 basepairs of either the upstream or downstream region of the gene to be disrupted.

The primers used to disrupt the enterotoxin genes are listed in Table 1. Electroporated cells were spread on Luria-Bertani agar plates containing kanamycin (10 µg/ml) or chloramphenicol (5 µg/ml) to select for antibiotic resistant transformants. Subsequently the antibiotic cassettes were removed from the *estA*, *estB* or *eltAB* mutants by transformation with pCP20. pCP20 shows temperature-sensitive replication and can be thermally induced to express Flippase recombination enzyme, which acts on the FRT sites flanking the resistance genes. To generate the double mutant strains GIS26*ΔestBΔeltAB* and GIS26*ΔestAΔestB:KAN*, the same method was used, starting from GIS26*ΔestB* or GIS26*ΔestA*, respectively. In GIS26*ΔestAΔestB:KAN* the kanamycin resistance gene is still present. In all mutant strains, the presence or absence of all 3 toxin genes was verified by PCR with primers chosen 100 to 150 basepairs upstream and downstream from the coding sequences.

**Table 1:** Primer sequences used for the deletion of enterotoxin genes in ETEC strain GIS26.

Target gene (accession nr)	Primer	Primer sequence^a^
*estA* (V00612.1)	P-STa-F	TCCGTTTAACTAATCTCAAATATCCGTGAAACAACATGACGGGAGGTAAC*TGTGTAGGCTGGAGCTGCTTC*
	P-STa-R	CAATACATATAATATAGAGGGAATCAAAATAAAGATTCCCTCTATGCTTT*CATATGAATATCCTCCTTAG*
*eltAB* (DQ778054)	P-LT-F	CGTTATCTTTTTCCGGATTGTCTTCTTGTATGATATATAAGTTTTCCTCG*TGTGTAGGCTGGAGCTGCTTC*
	P-LT-R	ACAGTAGTTGTTATATAGGCTCCTAGCATTAGACATGCTTTTAAAGCAAA*CATATGAATATCCTCCTTAG*
*estB* (M35586)	P-STb-F	CCCACTGGTATAAGTTTTATTGCTTATAGCAATAAGGTTGAGGTGATTTTTG*TGTAGGCTGGAGCTGCTTC*
	P-STb-R	ATGAAAAATTATTTTTGTGTATATGGTGCTGAATGCTATTGATAAATATA*CATATGAATATCCTCCTTAG*

a Sequence in italic targets the antibiotic resistance cassette to be amplified from a template, the other 50 bp are the regions upstream or downstream of the gene to be disrupted (method derived from [24]).

### Toxin Detection and Quantification

Different methods were used to verify absence of toxin production in the different mutant strains. Bacterial strains were grown overnight at 37°C in Casamino Acids-Yeast Extract (CA–YE) medium pH 8.2 while shaking. For the detection of LT 0.25% w/v glucose was added to the growth medium for maximum toxin secretion. Before harvesting the supernatants, OD values at 650 nm of all strains were adjusted to the same value with CA-YE medium. For filtration of the supernatants a 0.22 µm low protein-binding filter was used (Millipore, Massachusetts, USA).

LT was detected in filtered supernatant of polymyxin B-treated cultures by the commercial VET-RPLA kit (Oxoid, Hampshire, UK), a reversed passive latex agglutination test and quantified by a GM_1_ enzyme-linked immunosorbent assay (ELISA), using 100 µl of undiluted filtered supernatant [25]. The detection limit of the GM_1_ ELISA was 0.1 ng/ml.

STa secretion was demonstrated with two commercial competitive enzyme immunoassays (EIA) (Oxoid, Hampshire, UK and Bachem, Bubendorf, Switzerland), following manufacturer’s instructions. The assay provided by Bachem also allowed for quantification of the toxin (detection limit of 0.6 ng/ml).

STb secretion was detected by immunoblotting using a polyclonal rabbit anti-STb serum (Dr. J. Daniel Dubreuil). Briefly, filtered supernatant of the overnight cultures was boiled for 5 minutes in Laemmli sample buffer. Proteins were separated using a 10–20% Tris-Tricine gel (Bio-Rad, California, USA) and blotted onto a polyvinylidene fluoride membrane. Following overnight blocking the membrane was incubated with a 1/500 dilution of the STb antiserum. The secondary antibody was a swine anti-rabbit Ig labeled with horseradish peroxidase (Dako, Glostrup, Denmark). Enzymatic activity was revealed by enhanced chemiluminescence (ECL) using Pierce ECL Western Blotting Substrate (Thermofisher Scientific, Illinois, USA). A direct STb ELISA was also performed as previously described, using the polyclonal rabbit anti-STb serum from Dr. J. Daniel Dubreuil [26] for quantification (detection limit of ±80 ng/ml). Briefly, supernatant of the overnight cultures was filtered and two-fold dilution series in 0.1 M carbonate buffer pH 9.6 were coated overnight at 4°C on Maxisorp plates (Nunc, New York, USA). Subsequent incubation steps were; blocking for 2 h at 37°C with 3% gelatin, incubation for 1 h at 37°C with a 1/100 dilution in PBS containing 0.05% Tween® 20 of the anti-STb antibody, incubation for 1 h at 37°C with a 1/1000 dilution in PBS containing 0.05% Tween® 20 of swine anti-rabbit Ig labeled with horseradish peroxidase, incubation for 30 minutes at 37°C with a 2,2′-azino-bis(3-ethylbenzthiazoline-6-sulphonic acid) (ABTS) (Roche, Basel , Switzerland) solution containing H_2_O_2_. In between each incubation step, plates were washed with PBS containing 0.05% Tween® 20. The OD of the wells was measured at 405 nm.

### Anesthesia Protocol

Piglets were fasted overnight. The next morning, premedication was administered by intramuscular injection of 40 mg/kg azaperon (Janssen Animal Health, Beerse, Belgium) and 0.1 mg/kg morphine (Sterop, Brussels, Belgium). After 20 minutes, anesthesia was induced by IV injection of 2–4 mg/kg propofol (AST Farma, Oudewater, The Netherlands). After endotracheal intubation the piglets were kept under long-term anesthesia with a mixture of 1.5% isoflurane (Ecuphar, Oostkamp, Belgium) and 40% oxygen (Air Liquide, Luik, Belgium). Fentanyl (Janssen-Cilag, Beerse, Belgium) at a rate of 5–10 µg/kg/h was given IV as an analgesic. Hematocrit values (Hct) were assessed at regular timepoints and when they exceeded 35, 10–15 ml/kg/h ringer lactate (Baxter, Illinois, USA) was infused via the ear vein to prevent dehydration. Temperature, heart rate, oxygen saturation, expiratory CO_2_ and non-invasive blood pressure were monitored continuously.

### Surgery

The surgical and experimental procedures have previously been described in detail [21], [27]. In brief, the abdomen was opened at the linea alba and five small intestinal segments of 20 cm in length were made in the mid-jejunum, starting at a distance of 200 cm distal to the ligament of Treitz. These segments retained their vascularization and were cannulated with a rubber tube at the proximal and distal ends to inject and collect fluid respectively.

### Bacterial Inoculum

The GIS26 strain or its isogenic mutant strains were cultured for 16 h in Tryptone Soy Broth (Difco Laboratories, Bierbeek, Belgium), and bacteria were collected by spinning at 5000×g for 15 minutes. Subsequently, the bacteria were washed and resuspended in PBS at a concentration of 5×10^8^ bacteria per ml (OD_660_ of 0.5), as confirmed by counting CFU.

### Perfusion

SISP experiments were performed essentially as described by Nabuurs *et al*. [21]. Three piglets were used to compare the effect of GIS26 WT and four mutant strains on net absorption and host early immune responses (microarray analysis). In addition, five piglets were used to further investigate the role of STb on net absorption. In brief, fifteen minutes before perfusion, segments were injected with 5 ml bacterial inoculum (2.5×10^9^ CFU) or with PBS only (control). The position of the GIS26 mutants, the GIS26 wild type strain and PBS was randomized. Intestinal segments were perfused with 0.9% NaCl, supplemented with 0.1% glucose and 0.1% casamino acids. Each segment was perfused with 32 ml over 4 h by injecting 2 ml of perfusion fluid every 15 minutes whereafter piglets were euthanized with an overdose sodium pentobarbital (Kela Laboratoria, Hoogstraten, Belgium). Residual fluids in the segments were collected and in the three pigs used for microarray analysis, a small piece of tissue of each segment was sampled and frozen for RNA isolation. Net fluid absorption was calculated from the difference between the inflow and outflow divided by the surface area (length × circumference) of each segment.

### Isolation of Total RNA

Approximately 100 mg of frozen intestine was homogenized in 1 ml TRIzol® Reagent (Invitrogen, Merelbeke, Belgium) to extract total RNA. These homogenates were further purified using the RNeasy Mini Kit (Qiagen Benelux, Venlo, The Netherlands) with an on column DNase treatment (RNase-free DNase set, Qiagen Benelux). Spectrophotometric RNA quality control was done using Nanodrop® ND-1000 (Isogen Life Science, De Meern, The Netherlands) using only samples with a 260/280 ratio between 1.8-2.1 and 260/230 ratio between 1.5-2.0. RNA integrity was assessed using a Bioanalyser 2100 (Agilent, California, USA). A part of the isolated RNA was used for microarray analysis, and another part was for expression analysis of selected genes by PCR.

### Microarray Analysis

The Porcine Genome Array (Affymetrix, California, USA) was used containing 23,937 probe sets to interrogate 23,256 transcripts in pig, which represents 20,201 *Sus scrofa* genes. Per sample, an amount of 100 ng of total RNA spiked with bacterial RNA transcript positive controls (Affymetrix) was converted to double stranded cDNA in a reverse transcription reaction. Subsequently, the sample was converted and amplified to antisense cRNA and labeled with biotin in an *in vitro* transcription reaction. All steps were carried out according to the manufacturers protocol (Affymetrix). A mixture of purified and fragmented biotinylated cRNA and hybridisation controls (Affymetrix) was hybridized on Affymetrix GeneChip® Porcine Genome Arrays followed by staining and washing in a GeneChip® fluidics station 450 (Affymetrix) according to the manufacturer’s instructions. To assess the raw probe signal intensities, chips were scanned using a GeneChip® scanner 3000 (Affymetrix).

### Analysis of Microarray Data

R (version 2.11.1), a free software environment for statistical computing and graphics, was used in combination with the *affy* library (version 1.26.1) of BioConductor (www.bioconductor.org) to calculate the MAS 5.0 detection calls and the RMA [28] expression values. The MAS 5.0 detection calls were used to decide whether a signal was significantly above background.

For 5,781 probe sets, none of the signals had a present detection call and these were omitted from further analysis. Also the spot controls were removed prior to the analysis. A set of 18,246 probe sets was retained. The normalized intensity values of the different conditions were compared with the limma package (version 3.4.3, [29]) of Bioconductor. Hereto, a linear model with pig and treatment as factors was fitted. With this design, estimates for all effects of interest were obtained. These contrasts of interest were estimated and tested whether they were significantly deviating from 0 with a moderated t-statistic. Transcripts were selected based on the more stringent cut-off of the uncorrected P-values, i.e. P<0.001. This cut off on the P-values was combined with a cut-off on the fold-change of two (i.e., an absolute log2 ratio larger than 1).

To annotate the probes, the latest probe annotations (NetAffx annotation date 2008-12-01 and build 27) were applied. In addition the annotation described by Tsai *et al.* was used (http://www4.ncsu.edu/~stsai2/annotation/) [30].

### Quantitative Real-time PCR Analysis

Two µg of total RNA of each sample was converted to single stranded complementary DNA by reverse transcription (AMV-Reverse Transcriptase, Promega Benelux) with random priming.

Nine genes from the microarray analysis were selected for confirmation by quantitative real-time PCR. Intestinal housekeeping genes *ribosomal protein L4* (*RPL4*) and *glyceraldehyde-3-phosphate dehydrogenase* (*GAPDH*) were chosen after checking the expression stability of a set of five housekeeping genes using the Genorm software [31]. Primers for *RPL4*, *GAPDH*, *IL8*, *PAP* and *FABP2* (Table 2) were from a previous study [32]. The primers for *IL1A*, *IL17A*, *TLR4*, *MMP1*, *MMP3* and *CYP1A1* (Table 2) were designed using the Beacon Designer software (PREMIER Biosoft International, California, USA). To avoid contamination of genomic DNA the primers were chosen in different exons. Primer concentrations were tested during optimization reactions using pooled cDNA.

**Table 2:** Primer sequences used for qRT-PCR.

Symbol	Name	Probe set ID	Forward primer	Reverse primer
*RPL4*	Ribosomal protein L4	Ssc.12277.1.S1_at	GAGAAACCGTCGCCGAAT	GCCCACCAGGAGCAAGTT
*GAPDH*	Glyceraldehyde-3-phosphate dehydrogenase	Ssc.14942.1.S1_at	GGTCGGAGTGAACGGATTTG	ACTGTGCCGTGGAATTTGC
*IL1A*	Interleukin-1, alpha	Ssc.113.1.S1_at	TCCTGTGACTCTAAGAATCT	CCAGAAGAAGAGGAGACT
*IL8*	Interleukin-8	Ssc.658.1.S1_at	TCACAAGCTCCTAGGACCAGA	CAGAACTGCAGCCTCACAGA
*IL17A*	Interleukin-17, alpha	SscAffx.23.1.S1_at	CCCTCAGATTACTCCAAA	CCTTCAGCATTGATACAG
*PAP*	Pancreatitis-associated protein	Ssc.16470.1.S1_at	GAAGATTCCCCAGCAGACAC	AGGACACGAAGGATGCCTC
*FABP2*	Intestinal fatty acid binding protein	Ssc.16525.1.S1_at	TGAATCAGCTGGAGACTATGG	TTTACCACGTTAATACCCATTTTT
*TLR4*	Toll-like receptor 4	Ssc.12781.1.A1_at	TGAGTCATTTAGACAGCAATAGC	CCGTCAGTATCAAGGTGGAA
*MMP1*	Matrix metallopeptidase 1 (interstitial collagenase)	Ssc.16013.1.S1_at	GAGATTGCCGATAGAGATGAAG	ACTAGGGAAGCCAAAGGAT
*MMP3*	Matrix metallopeptidase 3 (stromelysin 1, progelatinase)	Ssc.15927.1.A1_at	GATGATGTGAATGGCATT	CTGAGGTGTAGATTCTGT
*CYP1A1*	Cytochrome P450 1A1	Ssc.208.1.S1_at	TGTGAACCAGTGGCAGAT	CATCGGCAGTGAGAAACC

Subsequently, quantitative real-time PCR (qRT-PCR) was performed for each primer set using the SYBR® Green PCR Master Mix (Applied Biosystems, California, USA) and 100 ng of template cDNA. A two-step program was run on the StepOnePlus real-time PCR system (Applied Biosystems). Thermal cycling conditions were 95°C for 10 min followed by 40 cycles of 95°C for 15 s and 60°C for 1 min. Melting curve analysis confirmed primer specificities. All reactions were run in triplicate and a standard curve for all genes, including housekeeping genes, was generated using serial dilutions of a pooled sample. PCR efficiency of 90-110% (3.2< slope >3.8) together with a correlation coefficient of >0.99 were accepted. Values for each target gene were normalized using the geometric mean of the expression of *RPL4* and *GAPDH*, according to the standard curve method for the analysis of the expression of the genes [33].

### Statistical Analysis

Graphpad Prism version 4.00 for Windows (GraphPad Software, San Diego California USA) was used to analyze STa EIA and perfusion experiments. STa EIA results and net absorption data of perfusion experiments were analyzed using one-way analysis of variance and the Bonferroni post-hoc test. Analysis on STb ELISA results was performed using Deltasoft Microplate analysis software (BioMetallics Incorporated, New York, USA).

The relationship between the levels of gene expression of selected genes, qRT-PCR versus microarray data, was determined by linear regression.

## Results

### *In vitro* Toxin Phenotype of Mutant Strains Differs from their Toxin Genotype

**Figure 1:**
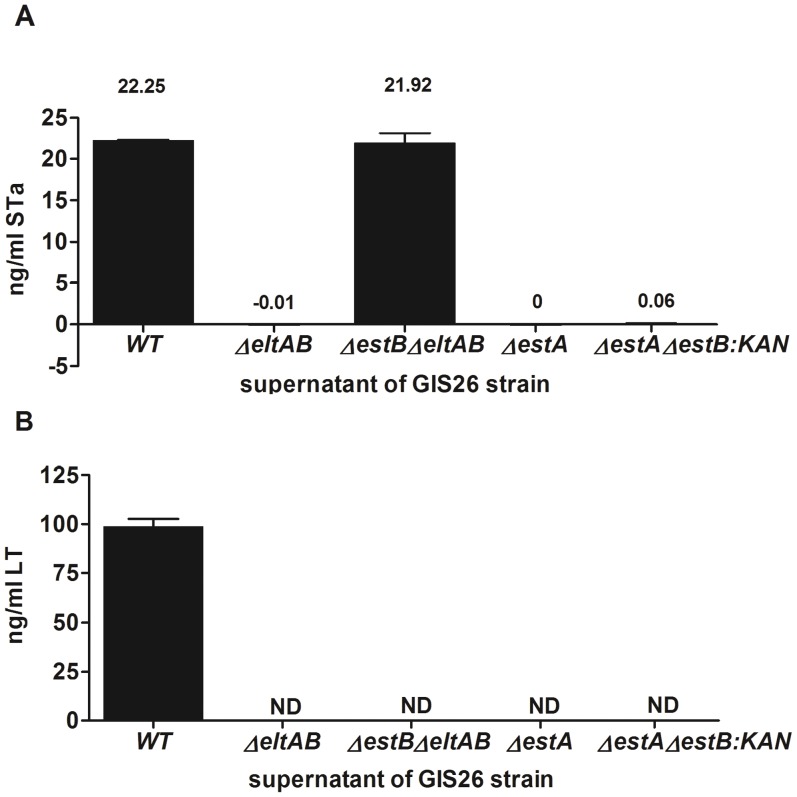
Quantitative detection of STa (A) and LT (B) expression by different isogenic ETEC strains following *in vitro* culture. Both toxins were detected by enzyme immunoassays. Mean values ± SD are shown. (A) Samples for STa were tested in triplicate in three independent experiments. (B) LT results are representative for 2 independent experiments. ND  =  below detection limit of 10 ng/ml. WT  =  wild type strain.

**Figure 2:**
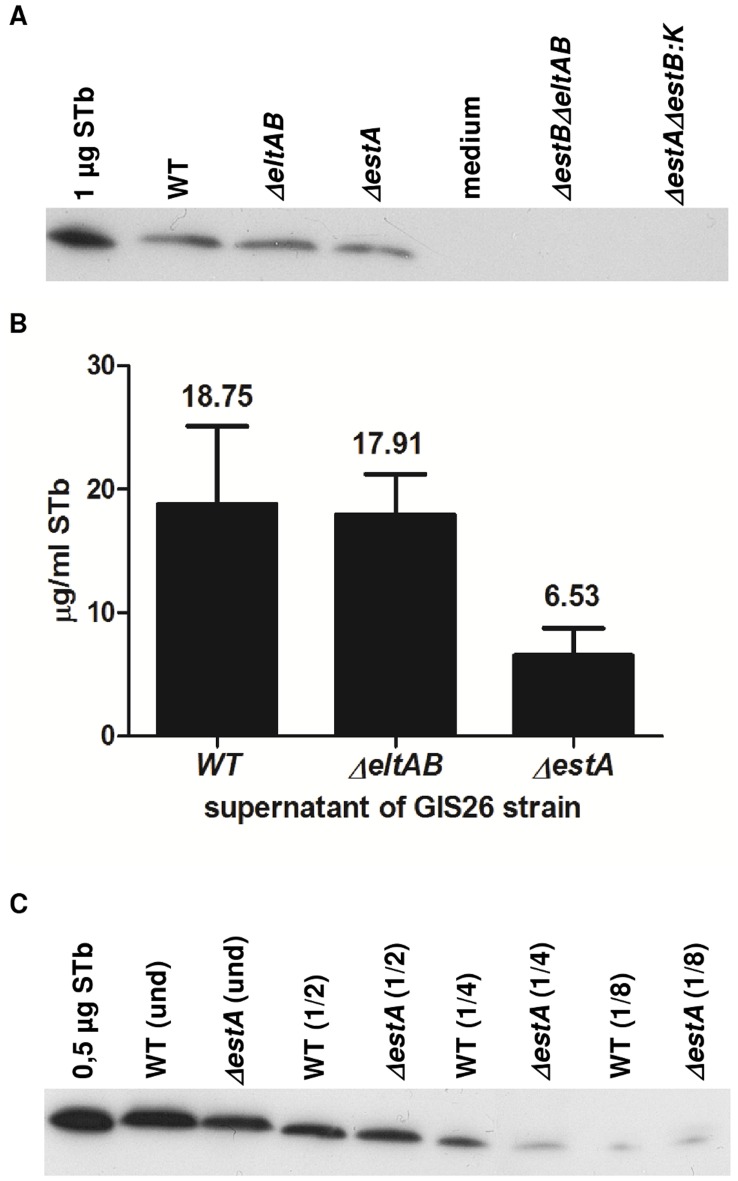
Detection of STb expression by different isogenic ETEC strains following *in vitro* culture. (A) STb detection with Western blotting. For every GIS26 mutant an equal amount of filtered supernatant was loaded (30 µl), data are representative for 3 independent experiments. (B) In the STb positive strains, STb was quantified by a direct ELISA. Mean values±SD are shown; results are representative for 3 independent experiments. (C) A difference in amount of STb produced between the wild type strain and the GIS26*ΔestA* mutant was also detected by Western blotting of different dilutions of the supernatant of both strains (20 µl per lane).

Absence of the targeted toxin genes was first verified by PCR and sequencing of the toxin genes for each of the generated mutant strains. Simultaneously the presence of the other wild type toxin gene(s) was also verified for each mutant (Table 3: genotype). Next, we compared *in vitro* production of the toxins between wild type and mutant strains. Both GIS26*ΔestA* and GIS26*ΔestAΔestB:KAN* lack production of STa as compared to GIS26 wild type and GIS26*ΔestBΔeltAB* (P<0.001) (Figure 1A). However, unexpectedly no STa was detectable in the GIS26*ΔeltAB* strain (P<0.001). This was confirmed with two different kits. The presence or absence of LT in supernatant of polymyxin B-lysed bacteria was verified five times with a non-quantitative method. Subsequently, these results were confirmed through detection of LT in the normal culture supernatant by a quantitative method (Figure 1B). Only the wild type GIS26 produced detectable amounts of LT. In Figure 2A, the results of STb detection in culture supernatant are presented. Purified STb was used as a positive control. The wild type strain and both GIS26*ΔestA* and GIS26*ΔeltAB* mutant strains showed a clear band for STb. In contrast, no STb could be detected in the supernatant of GIS26*ΔestBΔeltAB* and GIS26*ΔestAΔestB:KAN* mutants. The supernatant of these negative strains was 10x concentrated by trichloroacetic acid precipitation but also in these samples there was no detection of STb (data not shown). Quantifying the amount of STb by direct ELISA (Figure 2B) revealed a 3-fold reduction in amount of STb for GIS26*ΔestA* as compared to the wild type strain. This was confirmed by Western blot when equal amounts of the supernatant of both the wild type strain and the GIS26*ΔestA* mutant were diluted 4 times (Figure 2C).

**Table 3:** Enterotoxin genotype and phenotype of GIS26 mutants used in this study.

	Genotype^a^			Phenotype^b^			
GIS26 strain	*estA*	*estB*	*eltAB*	STa	STb	LT	new strain designation
wild type	+	+	+	+	+	+	GIS26(STa^+^ STb^+^ LT^+^)
*ΔeltAB*	+	+	–	**−**	+	–	GIS26(STa^−^ STb^+^ LT^−^)
*ΔestBΔeltAB*	+	–	–	+	**−**	–	GIS26(STa^+^ STb^–^ LT^−^)
*ΔestA*	–	+	+	–	**+**	**−**	GIS26(STa^−^ STb^low^ LT^−^)
*ΔestAΔestB:KAN*	–	–	+	–	–	**−**	GIS26(STa^−^ STb^–^ LT^−^)

a Genotype was assessed by PCR and sequencing.

b Phenotype was determined by detection of the different enterotoxins in culture supernatant as shown in Figure 1 and Figure 2.

Conclusions from these data are summarized in Table 3 in which a new strain designation for every mutant is introduced based on the phenotype. To avoid confusion, this new designation was used throughout the rest of this manuscript.

### STb Seems to Play an Important Role in the Induction of Small Intestinal Secretion

Strains were administered *in vivo* in small intestinal segments using the SISP technique and the capacity of the mutants to induce a secretory response was compared to the wild type strain. All used piglets were F4 receptor positive as determined by an *in vitro* villous adhesion assay [23]. In each piglet, an uninfected (PBS control) segment, a wild type GIS26-infected segment and segments infected with the different mutant strains were present. The segments were perfused during 4 h and net absorption was calculated.

**Figure 3:**
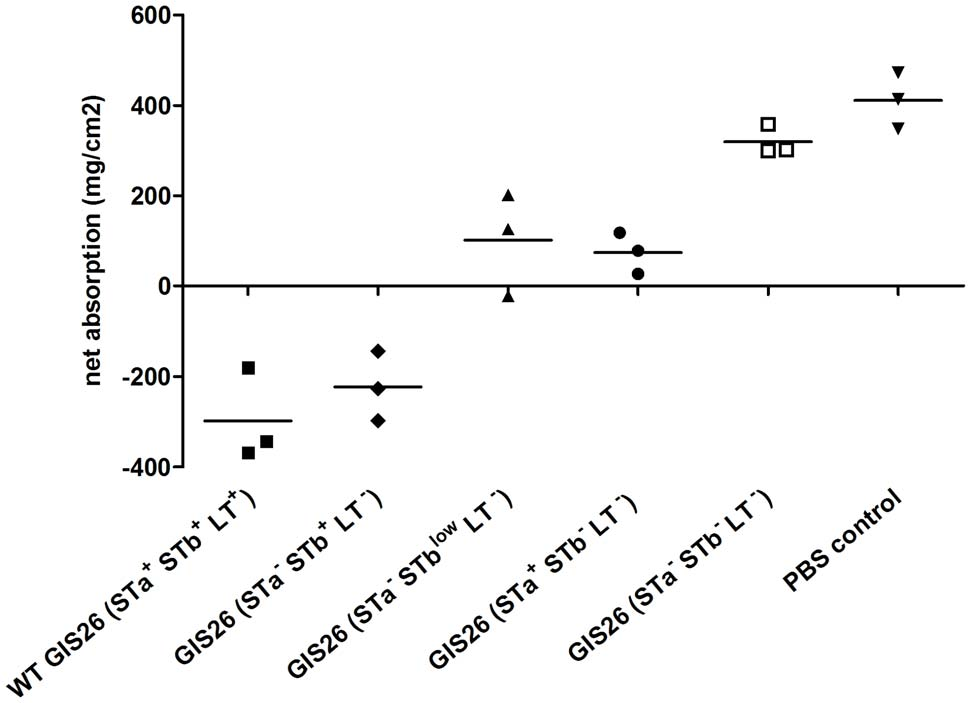
Effect of wild type and mutant GIS26 ETEC strains on net fluid absorption (mg/cm^2^) in 4 h-infected jejunal segments. Individual data per piglet and the mean from 3 individual experiments are presented.

In a first set of experiments, the effect of the wild type GIS26 strain on net absorption was compared to the effects of mutant strains GIS26 (STa^−^ STb^+^ LT^−^), GIS26 (STa^−^ STb^low^ LT^−^), GIS26 (STa^+^ STb^–^ LT^−^) and GIS26 (STa^−^ STb^−^ LT^−^) (Figure 3). For all three piglets the PBS segments showed net absorption while the wild type GIS26 (STa^+^ STb^+^ LT^+^)-infected segments all showed net secretion (P<0.001). As expected, the mutant strain GIS26 (STa^−^ STb^−^ LT^−^) that did not express enterotoxins was no longer able to reduce net absorption and values were comparable with the PBS group. All other mutant strains significantly reduced net absorption when compared to the PBS segments. GIS26 (STa^−^ STb^+^ LT^−^), producing only STb was as potent in inducing net secretion as the wild type strain, indicating an important role for STb in the induction of secretory responses by the GIS26 ETEC strain in intestinal segments of 5-week-old piglets. Indeed, the GIS26 (STa^−^ STb^low^ LT^−^) strain, producing only low amounts of STb showed a significant higher absorption compared to the GIS26 (STa^−^ STb^+^ LT^−^) mutant (P<0.01), indicating that also the amount of produced STb is important. The strain producing only STa, GIS26 (STa^+^ STb^−^ LT^−^) induced a similar effect on fluid secretion as the strain secreting only a low amount of STb, whereas a significantly lower effect occurred on net absorption than the GIS26 (STa^−^ STb^+^ LT^−^) or the wild type strain (P<0.01), indicating that STa only played a minor role in the reduction of net absorption by the wild type strain.

**Figure 4:**
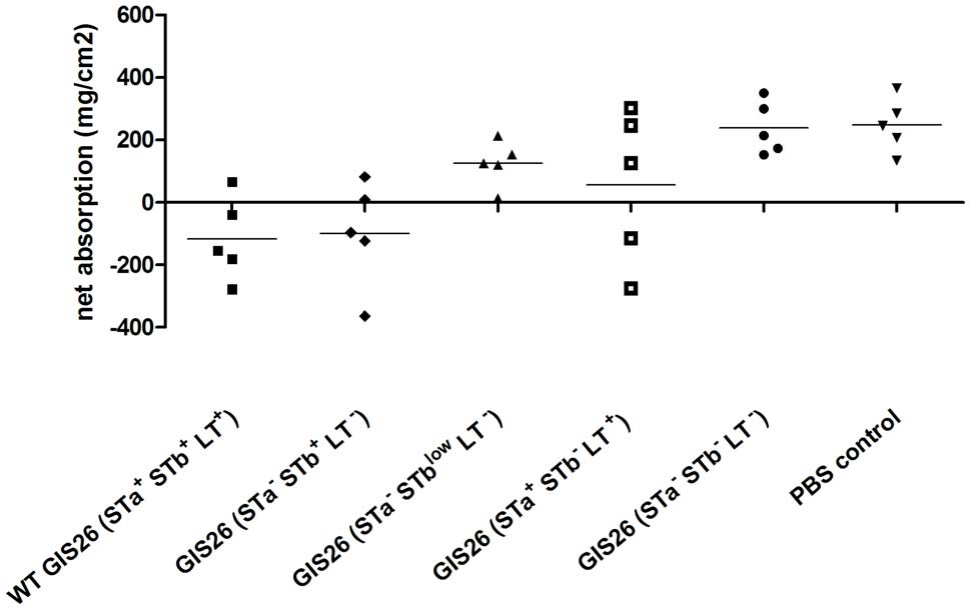
Effect of STb deletion in GIS26 ETEC strain on net fluid absorption (mg/cm^2^) in 4 h-infected jejunal segments. Individual data per piglet and the mean from 5 individual experiments are presented.

To further confirm the role for STb in the induction of secretion by the GIS26 ETEC strain we performed five additional SISP experiments with another mutant strain, GIS26 (STa^+^ STb^−^ LT^+^) – genotype GIS26*ΔestB* - that produces no STb but normal levels of LT and STa. This phenotype was confirmed with the same methods as for the other mutants (data not shown). The previous effects on net absorption for GIS26 wild type, GIS26 (STa^−^ STb^+^ LT^−^), GIS26 (STa^−^ STb^low^ LT^−^) and GIS26 (STa^−^ STb^−^ LT^−^) are confirmed in these 5 extra piglets (Figure 4). The absence of STb in GIS26 (STa^+^ STb^−^LT^+^) seems to have a variable effect on the ability of this mutant strain to reduce net absorption. In 2 piglets there was no difference between wild type GIS26 (STa^+^ STb^+^ LT^+^)-infected and GIS26 (STa^+^ STb^−^ LT^+^)-infected segments whereas in the other 3 piglets there was a clear and even significant difference (P<0.001). Altogether results indicate that STb is important in the induction of secretory responses by the GIS26 ETEC strain in intestinal segments of 5-week-old piglets but that presence of STa and LT may be able to compensate for the lack of STb, especially in piglets where the wild type ETEC strain has a strong secretory effect.

### Microarray Analysis

#### ETEC regulates gene expression of several porcine genes important in inflammatory responses

Intestinal cDNA isolated from mock (PBS)-infected segments was compared with cDNA from wild type ETEC-infected segments, to detect differences in gene expression, 4 h after infection. The difference in gene expression between mock-infected and ETEC-infected segments was determined as the statistical mean of three piglets, indicating the average differential expression. In the latter comparison, 153 transcripts were down-regulated and 157 up-regulated (Table 4). Because of the large number of differentially expressed transcripts, a more stringent cut-off, log-ratio <−2 and log-ratio >2 was used, after which 15 ETEC down-regulated (PBS vs. ETEC) and 23 ETEC up-regulated (ETEC vs. PBS) transcripts remained (Table 5 and Table 6).

**Table 4:** Summary of differentially expressed probe sets of all mutant strains and control versus wild type ETEC strain.

	Uncorrected P value <0.001			
	log-ratio <−1	log-ratio >1	log-ratio <−2	log-ratio >2
PBS vs. GIS26 (STa^+^ STb^+^ LT^+^)	153	157	23	15
GIS26 (STa^−^ STb^+^ LT^−^) vs. GIS26 (STa^+^ STb^+^ LT^+^)	0	0		
GIS26 (STa^+^ STb^−^ LT^−^) vs. GIS26 (STa^+^ STb^+^ LT^+^)	0	0		
GIS26 (STa^−^ STb^low^ LT^−^) vs. GIS26 (STa^+^ STb^+^ LT^+^)	20	23		
GIS26 (STa^−^STb^−^ LT^−^) vs. GIS26 (STa^+^ STb^+^ LT^+^)	27	27		

Most transcripts down-regulated by ETEC (Table 5) are not associated with immune responses, the majority of genes has a specific function in the intestinal metabolism (*PCK1, PTPRR, SLC25A27, PRR15, PPARGC1A, ATP10D, CDC10, KIAA1468, GPT2, PHLPPL*) or in transport of fluids and electrolytes (*KCNJ13, AQP8, ATG10, APOC3*). Another transporter SLC26A3 is up-regulated by ETEC (Table 6). This protein is functionally coupled to CFTR (cystic fibrosis transmembrane regulator) and to NHE3 (Na/H exchanger-isoform 3), which are both involved in the secretory pathway of LT, STa and STb [34], [35].

**Table 5:** Transcripts down-regulated by ETEC. Microarray data expressed as a log2 fold-change of PBS versus ETEC-infected small intestinal segments at 4 h (for full list see Table S1).

Probe Set ID	Log2 ratio	Gene symbol	Gene title	Tentative function (UniprotKB)
Ssc.22959.1.S1_at	**3.96**	*PCK1*	Phosphoenolpyruvate carboxykinase	Gluconeogenesis
Ssc.21194.1.S1_at	**3.46**	*PTPRR*	Receptor-type protein-tyrosine phosphatase R precursor	Hydrolase, protein phosphatase, receptor
Ssc.18284.1.A1_at	**3.41**	*KCNJ13*	Inward rectifier potassium channel 13	Voltage-gated channel, potassium transport
Ssc.20419.1.S1_at	**2.71**	*SLC25A27*	Mitochondrial uncoupling protein 4	Transport (transmembrane), binding
Ssc.11487.1.A1_at	**2.66**	*PRR15*	Proline-rich protein 15	Developmental protein
Ssc.18488.1.S1_at	**2.59**	*AQP8*	Aquaporin 8	Transport
Ssc.29525.1.A1_at	**2.37**	*ATG10*	APG10 autophagy 10-like [*H. sapiens*]	Ligase, autophagy, protein transport, transport, Ubl conjugation pathway
Ssc.16864.1.S1_at	**2.37**	*PPARGC1A*	Peroxisome proliferator activated receptor gamma coactivator 1 alpha	Transcription, transcription activator
Ssc.9238.1.A1_at	**2.27**	*ATP10D*	Potential phospholipid-transporting ATPase VD	Hydrolase
Ssc.7301.1.A1_at	**2.22**	*CDC10*	Septin 7 (CDC10 protein homolog)	Cytokinesis, mitosis, cell cycle
Ssc.7991.1.A1_at	**2.16**	*KIAA1468*	Protein *KIAA1468*	Binding
Ssc.7458.1.A1_at	**2.15**	*GPT2*	Alanine aminotransferase 2 [*H. sapiens*]	Aminotransferase, transferase
Ssc.4724.1.S1_at	**2.14**	*PHLPPL*	PH domain leucine-rich repeat-containing protein phosphatase 2	Protein binding, catalytic activity
Ssc.20419.2.S1_at	**2.02**	*SLC25A27*	Mitochondrial uncoupling protein 4	Transport (transmembrane), binding
Ssc.1039.1.S1_at	**2.00**	*APOC3*	Apolipoprotein C-III precursor	Transport, G-protein coupled receptor protein signaling pathway

The selection criteria to define a transcript as differentially regulated were: an absolute log2 ratio equal or larger than 2, a MAS 5.0 present detection call and an uncorrected P value of <0.001.

In contrast, immunomodulatory genes are abundantly present in the list of 23 transcripts up-regulated after ETEC infection (Table 6). Among them, interleukin 1 (*IL1A* and *IL1B*), the interleukin 1 receptor antagonist (*IL1RN*) and interleukin 17 (*IL17A*), three cytokines with a known function in inflammatory responses, and *DUOX2* that plays a role in the signaling pathway of these cytokines. Furthermore, the genes *MMP1* and *MMP3* belonging to the family of matrix metalloproteinases (MMP) have been described to regulate various aspects of inflammation and immunity by acting on pro-inflammatory cytokines, chemokines and other proteins [36]. Also interesting is pancreatitis associated protein (PAP), alias *REG3A*, which has anti-bacterial and anti-inflammatory properties [37], [38], [39]. PAP, a marker for pancreatitis, is also expressed in Paneth cells [40], pig small intestine [41], and human colon where it is up-regulated after inflammation [42]. Another up-regulated gene, with a central role in the activation of inflammation, is the ectoderm-neural cortex-1 protein (*ENC1*), involved in the ubiquitin l conjugation pathway [43].

**Table 6:** Transcripts up-regulated by ETEC. Microarray data expressed as a log2 fold-change of ETEC-infected versus PBS treated small intestinal segments at 4 h (for full list see Table S1).

Probe Set ID	Log2ratio	Genesymbol	Gene title	Tentative function (UniprotKB)
Ssc.15927.1.S1_at	**4.18**	*MMP3*	Stromelysin-1 precursor; Matrix metalloproteinase-3	Proteolysis, metalloendopeptidase activity
Ssc.15927.2.S1_at	**4.16**	*MMP3*	Stromelysin-1 precursor; Matrix metalloproteinase-3	Proteolysis, metalloendopeptidase activity
SscAffx.23.1.S1_at	**3.68**	*IL17A*	Interleukin-17 precursor; Cytotoxic T lymphocyte-associated antigen 8	Cytokine, inflammatory response
Ssc.16470.1.S1_a_at	**3.10**	*PAP (REG3A)*	Pancreatitis-associated protein 1 precursor	Acute phase response, inflammatory response
Ssc.6189.1.A1_at	**2.90**	*SLC7A11*	Cystine/glutamate transporter	Response to toxin, transport
Ssc.15927.2.A1_at	**2.90**	*MMP3*	Stromelysin-1 precursor; Matrix metalloproteinase-3	Proteolysis, metalloendopeptidase activity
Ssc.17573.1.S1_at	**2.72**	*IL1B*	Interleukin-1 beta precursor	Inflammatory response, cytokine, pyrogen
Ssc.29329.1.A1_at	**2.71**	*DCHS2*	Dachsous 2 isoform 1 [*H. sapiens*]	Cell adhesion, calcium ion binding
Ssc.113.1.S2_at	**2.68**	*IL1A*	Interleukin-1 alpha precursor	Inflammatory response, cytokine, pyrogen
Ssc.15601.1.A1_s_at	**2.65**	*IL1B*	Interleukin-1 beta precursor	Inflammatory response, cytokine, pyrogen
Ssc.24966.1.S1_at	**2.47**	*NP*	Purine nucleoside phosphorylase	Glycosyltransferase, transferase
Ssc.30277.1.A1_at	**2.46**	*SLC26A3*	Chloride anion exchanger	Antiport, transport (excretion)
Ssc.18918.1.A1_at	**2.43**	*GPX2*	Glutathione peroxidase-gastrointestinal	Oxidoreductase, peroxidase, response tooxidative stress
Ssc.29281.1.A1_at	**2.35**	*SLC7A11*	Cystine/glutamate transporter	Response to toxin, transport
Ssc.33.1.S1_at	**2.33**	*DUOX2*	Dual oxidase 2 precursor [*H. sapiens*]	Oxidoreductase, peroxidase, cytokine-mediatedsignaling pathway
Ssc.113.1.S1_at	**2.30**	*IL1A*	Interleukin-1 alpha precursor	Inflammatory response, cytokine, pyrogen
Ssc.11609.1.A1_at	**2.28**	*ASNS*	Asparagine synthetase	Ligase
Ssc.19907.1.S1_at	**2.22**	*F3*	Tissue factor precursor	Blood coagulation
Ssc.12431.1.A1_at	**2.11**	*MYO5B*	Myosin Vb	Protein transport
Ssc.18603.1.A1_at	**2.07**	*G0S2*	Putative lymphocyte G0/G1 switch protein 2	Cell cycle
Ssc.16013.1.S1_at	**2.05**	*MMP1*	Interstitial collagenase precursor; Matrixmetalloproteinase-1	Proteolysis, metalloendopeptidase activity
Ssc.30857.1.S1_at	**2.05**	*ENC1*	Ectoderm-neural cortex-1 protein	Ubl conjugation pathway
Ssc.16250.1.S2_at	**2.01**	*IL1RN*	Interleukin-1 receptor antagonist protein precursor	Cytokine activity, interleukin-1 receptorantagonist activity, immune response,inflammatory response

The selection criteria to define a transcript as differentially regulated were: an absolute log2 ratio equal or larger than 2, a MAS 5.0 present detection call and an uncorrected P value of <0.001.

#### Microarray analysis of mutant ETEC - versus wild type ETEC- infected jejunum suggests a role for STb in ETEC-induced immune responses

To reveal an influence of the different enterotoxins on innate immune responses, the differential transcriptional regulation between wild type ETEC strain GIS26 and its isogenic mutants was analyzed in the microarray study. This study was performed with RNA of the three pigs in Figure 3.

The mutant strain, GIS26 (STa^−^ STb^+^ LT^−^), produced no STa and LT but showed normal STb levels as compared to the wild strain GIS26 (STa^+^ STb^+^ LT^+^) (Figure 1 and Figure 2). When microarray results from this mutant were compared to GIS26 (STa^+^ STb^+^ LT^+^) no differential transcripts were reported (Table 4). This result is in agreement with the secretory responses, where no significant difference could be detected between this mutant and the wild type strain (Figure 3 and Figure 4).

Another mutant, GIS26 (STa^+^ STb^−^ LT^−^) expressing only STa (Figure 1 and Figure 2), was also compared to GIS26 (STa^+^ STb^+^ LT^+^). Again no differential gene expression was revealed (Table 4). As this mutant strain showed a significant loss in capacity to reduce net absorption as compared to the wild type strain (Figure 3), this result indicates that STa can compensate for the absence of STb (and LT) in the activation of innate immune responses but not for the induction of secretion.

The third mutant GIS26 (STa^−^ STb^low^ LT^−^) only expressed reduced amounts of STb *in vitro* (Figure 1 and Figure 2), and when compared to the wild type strain, 43 transcripts were found to be differentially regulated of which 20 down-regulated (up-regulated in the wild type ETEC strain), and 23 up-regulated (Table 4).

When the mutant strain GIS26 (STa^−^ STb^−^ LT^−^), which expressed no enterotoxins at all, was compared to GIS26 (STa^+^ STb^+^ LT^+^), in total 54 transcripts were differentially regulated. Twenty-seven genes were down-regulated (up-regulated in the wild type ETEC strain), and 27 up-regulated (Table 4).

The differentially expressed transcripts were subdivided into five groups based on the presence or absence (up-regulated or down-regulated) in each of the three comparisons listed in Tables 7, 8, 9, 10 and 11.

In the first group, transcripts present in all three of the comparisons listed were found. The genes of group I (Table 7), up-regulated by ETEC, are probably regulated by the heat-stable enterotoxins expressed, since no differences in gene expression are found with mutants that still express STa or normal levels of STb but that lack LT. Among those *SLC26A3*, *IL1A* and *MMP3* were found. It can be speculated that high levels of STb or STa are important in the induction of these immune genes.

**Table 7:** Microarray data expressed as a log2 ratio of PBS and mutant ETEC-infected versus wild type ETEC-infected (WT) small intestinal segments at 4 h (Group I, transcripts in common for the three comparisons where differential regulation was found).

Probe Set ID	Log2 ratio			Gene symbol	Gene title	Tentative function (UniprotKB)
	GIS26 (STa^−^ STb^low^ LT^−^)/WT	GIS26 (STa^-^ STb^-^ LT^−^)/WT	PBS/WT			
Ssc.22959.1.S1_at	**2.83**	**3.24**	**3.96**	*PCK1*	Phosphoenolpyruvatecarboxykinase	Gluconeogenesis
Ssc.20419.1.S1_at	**1.52**	**1.86**	**2.71**	*SLC25A27*	Mitochondrialuncoupling protein 4	Transport (transmembrane), binding
Ssc.29525.1.A1_at	**1.62**	**1.88**	**2.37**	*ATG10*	APG10 autophagy10-like [*H. sapiens*]	Ligase, autophagy, protein transport, transport, Ubl conjugation pathway
Ssc.16864.1.S1_at	**1.42**	**1.44**	**2.37**	*PPARGC1A*	Peroxisome proliferatoractivated receptor gammacoactivator 1 alpha	Transcription, transcription activator
Ssc.7301.1.A1_at	**1.75**	**1.81**	**2.22**	*CDC10*	Septin 7	Cytokinesis, mitosis, cell cycle
Ssc.5000.1.A1_at	**1.23**	**1.32**	**1.85**	*ERBB2*	Receptor protein-tyrosinekinase erbB-2 precursor	Activator, kinase, receptor, transferase, tyrosine-protein kinase
Ssc.298.1.S1_at	**1.78**	**1.53**	**1.85**	*PRSS7*	Enteropeptidaseprecursor (Enterokinase)	Hydrolase, protease, serine protease
Ssc.14573.1.S1_at	**1.01**	**1.08**	**1.77**	*EYA2*	Eyes absenthomolog 2	Activator, chromatin regulator, developmental protein, hydrolase, protein phosphatase, transcription regulation
Ssc.10602.1.A1_at	**1.08**	**1.15**	**1.59**	*FLRT3*	Leucine-rich repeattransmembraneprotein FLRT3 precursor	Cell adhesion
Ssc.20832.1.S1_at	**1.26**	**1.24**	**1.55**	*SCTR*	Secretin receptorprecursor	G-protein coupled receptor, receptor, transducer
Ssc.16538.1.S1_at	**1.37**	**1.21**	**1.55**	*C1orf168*	–	–
Ssc.18915.1.A1_at	**1.23**	**1.3**	**1.54**	*ZC3H11A*	Zinc finger CCCHdomain-containingprotein 11A	Nucleic acid-, zinc ion-, protein binding
Ssc.27422.1.A1_at	**1.19**	**1.19**	**1.52**	*ACBD5*	acyl-Coenzyme A bindingdomain containing 5 [*H. sapiens*]	Transport
Ssc.17849.1.A1_at	**1.44**	**1.20**	**1.51**	*SLC30A10*	Solute carrier family 30;zinc transporter 8[*H. sapiens*]	Ion tranport, transport, zinc transport
Ssc.208.1.S1_at	**1.32**	**1.3**	**1.46**	*CYP1A1*	CytochromeP450 1A1	Monooxygenase, oxidoreductase
Ssc.7116.1.A1_at	**1.18**	**1.25**	**1.39**	*NT5C3*	5-nucleotidase;pyrimidine 5-nucleotidase [*H. sapiens*]	Hydrolase, transferase
Ssc.10703.1.A1_at	**1.52**	**1.4**	**1.02**	*SLC25A27*	Mitochondrialuncoupling protein 4	Transport (transmembrane), binding
Ssc.26709.1.S1_at	**−1.11**	**−1.22**	**−1.12**	*GPR183*	EBV-inducedG protein-coupledreceptor 2	Adaptive immunity, immunity, humoral immune response
Ssc.3509.1.S1_at	**−1.18**	**−1.14**	**−1.3**	*HK2*	Hexokinase,type II	Kinase, transferase
Ssc.11194.1.S1_at	**−1.18**	**−1.28**	**−1.32**	*PLAU*	Urokinase-typeplasminogen activatorprecursor	Blood coagulationn fibrinolysis, plasminogen activation
Ssc.18603.1.A1_at	**−1.57**	**−1.80**	**−2.07**	*G0S2*	Putative lymphocyte G0/G1switch protein 2	Cell cycle
Ssc.12431.1.A1_at	**−2.4**	**−2.46**	**−2.11**	*MYO5B*	Myosin Vb	Protein transport
Ssc.113.1.S1_at	**−1.79**	**−1.77**	**−2.3**	*IL1A*	Interleukin-1 alphaprecursor	Inflammatory response, cytokine, pyrogen
Ssc.30277.1.A1_at	**−1.79**	**−1.88**	**−2.46**	*SLC26A3*	Chloride anionexchanger	Antiport, transport (excretion)
Ssc.113.1.S2_at	**−1.69**	**−1.82**	**−2.68**	*IL1A*	Interleukin-1 alphaprecursor	Inflammatory response, cytokine, pyrogen
Ssc.15927.1.S1_at	**−2.3**	**−2.28**	**−4.18**	*MMP3*	Stromelysin-1precursor; matrixmetalloproteinase-3	Proteolysis, metalloendopeptidase activity

The selection criteria to define a transcript as differentially regulated were: an absolute log2 ratio equal or larger than 1, a MAS 5.0 present detection call and an uncorrected P value of <0.001.

In group II (Table 8) the retrieved genes were differentially regulated by both mutant strains with no LT, no STa and no or weak STb expression but not found in the PBS versus wild type strain comparison. Only three genes were left, namely *SERPINE1*, *TLR4*, and *SLC2A14* (down-regulated). Of these three, the serine protease inhibitor SERPINE1 and the Toll-like receptor for LPS (TLR4) have a well-known function in the immune/inflammatory response.

**Table 8:** Microarray data expressed as a log2 fold-change of PBS and mutant ETEC-infected versus wild type ETEC-infected (WT) small intestinal segments (Group II, transcripts differentially regulated by both mutant ETEC strains).

Probe Set ID	Log2 ratio			Gene symbol	Gene title	Tentative function
	GIS26 (STa^−^ STb^low^ LT^−^) /WT	GIS26 (STa^−^ STb^−^ LT^−^) /WT	PBS/WT			
Ssc.9781.1.S1_at	**−1.36**	**−1.25**	−1.11	*SERPINE1*	Plasminogen activator inhibitor-1 precursor	Plasminogen activation, cellular response to LPS, defense response to Gram-negative bacterium, positive regulation of IL-8 production, positive regulation of leukotriene production involved in inflammatory response
Ssc.12781.1.A1_at	**−1.16**	**−1.26**	−0.94	*TLR4*	Toll-like receptor 4 precursor	Immunity, inflammatory response, innate immunity, lipopolysaccharide receptor activity
Ssc.1674.1.A1_at	**−1.09**	**−1.08**	−0.74	*SLC2A14*	Glucose transporter 14 [H. sapiens]	Developmental protein, glucose transmembrane transporter activity

The selection criteria to define a transcript as differentially regulated (indicated in bold) were: an absolute log2 ratio equal or larger than 1, a MAS 5.0 present detection call and an uncorrected P value of <0.001. Transcripts not in bold are not differentially regulated as they do not meet these strict requirements.

Group III consists of those transcripts present in PBS versus wild type but differentially regulated in only one of both mutant strains. Group III (Table 9) therefore can be subdivided into two categories. The first one includes six transcripts present both in GIS26 (STa^−^ STb^low^ LT^−^) versus wild type and PBS versus wild type comparisons. Most notable is the 2-fold down-regulation of *MMP3* in two transcripts absent from GIS26 (STa^−^ STb^−^ LT^−^) versus wild type, although *MMP3* seemed equally down-regulated in both mutants when comparing another transcript (Ssc.15927.1.S1_at) (Group I, Table 7). In mutant GIS26 (STa^−^ STb^−^ LT^−^) the P-values for the two other transcripts (Ssc.15927.2.A1_at and Ssc.15927.2.S1_at) were at the borderline (Table S1). However, when less stringent P-values were applied these transcripts were also retrieved as differential expressed in mutant GIS26 (STa^−^ STb^−^ LT^−^) compared to the wild type strain. The second category within Group III (Table 9), represents 24 transcripts in common for both GIS26 (STa^−^ STb^−^ LT^−^) versus wild type and PBS versus wild type strain. The larger number of genes in common can be explained by the complete absence of enterotoxins in the mutant strain, by which its effect on net absorption is quite similar to PBS. There is no clear difference in the log2 fold change between these two groups except the fact that transcripts in GIS26 (STa^−^STb^−^ LT^−^) versus wild type ETEC have a lower expression as compared to PBS versus the wild type strain. This may be due to the presence of LPS and/or other metabolites in the mutant-infected segments. However, the fact that some genes involved in immune regulation like *IL1B*, and *IL17A*, are listed here and not with the GIS26 (STa^−^ STb^low^ LT^−^) mutant is interesting. It suggests that STb can regulate these genes, since this toxin is completely absent in the mutant strain GIS26 (STa^−^ STb^−^ LT^−^).

**Table 9:** Microarray data expressed as a log2 fold-change of PBS and mutant ETEC-infected versus wild type ETEC-infected (WT) small intestinal segments at 4 h (Group III, transcripts differentially regulated in the PBS/WT comparison and in one of both mutant strains).

Probe Set ID	Log2 ratio			Genesymbol	Gene title	Tentative function (UniprotKB)
	GIS26 (STa^−^STb^low^ LT^−^) /WT	GIS26 (STa^−^STb^−^ LT^−^)/WT	PBS/WT			
Ssc.7991.1.A1_at	**1.84**	1.47	**2.16**	*KIAA1468*	–	Binding
Ssc.1039.1.S1_at	**1.74**	1.47	**2.00**	*APOC3*	Apolipoprotein C-III precursor	Transport, G-protein coupled receptor protein signaling pathway
Ssc.24638.1.S1_at	**1.42**	1.27	**1.67**	*PRLR*	Prolactin receptor precursor	Receptor, T cell activation
Ssc.1147.1.A1_at	**−1.18**	−0.95	**−1.76**	*LPL*	Lipoprotein lipase precursor	Lipid degradation, hydrolase
Ssc.15927.2.A1_at	**−1.72**	−1.58	**−2.90**	*MMP3*	Stromelysin-1 precursor; matrix metalloproteinase-3	Proteolysis, metalloendopeptidase activity
Ssc.15927.2.S1_at	**−2.28**	−2.23	**−4.16**	*MMP3*	Stromelysin-1 precursor; matrix metalloproteinase-3	Proteolysis, metalloendopeptidase activity
Ssc.18284.1.A1_at	2.45	**2.86**	**3.41**	*KCNJ13*	Inward rectifier potassium channel 13	Voltage-gated channel, potassium transport
Ssc.9238.1.A1_at	1.08	**1.64**	**2.27**	*ATP10D*	Potential phospholipid-transportingATPase VD	Hydrolase
Ssc.20419.2.S1_at	1.11	**1.86**	**2.02**	*SLC25A27*	Mitochondrial uncoupling protein 4	Transport (transmembrane), binding
Ssc.22210.2.S1_at	1.34	**1.45**	**1.99**	*MTHFD2L*	Similar to Bifunctional methylenetetrahydrofolatedehydrogenase/cyclohydrolase[*H. sapiens*]	Hydrolase, oxidoreductase
Ssc.27342.1.S1_at	1.11	**1.34**	**1.85**	*ONECUT2*	–	Transcriptional activator
Ssc.27502.1.S1_at	1.38	**1.73**	**1.85**	*ITGB8*	Intergrin beta-8	Integrin, receptor, cell adhesion
Ssc.24037.1.S1_at	1.14	**1.44**	**1.83**	*UBE2U*	–	Ubl conjugation pathway
Ssc.28515.1.S1_at	1.13	**1.25**	**1.61**	*USP2*	Ubiquitin carboxyl-terminal hydrolase 2	Cell cycle, Ubl conjugation pathway
Ssc.2132.1.S1_a_at	1.03	**1.16**	**1.53**	*RPS6KA5*	Ribosomal protein S6 kinase alpha 5	Serine/threonine-protein kinase, transferase, response to stress and external stimulus
Ssc.30861.1.A1_at	0.93	**1.11**	**1.33**	*FLRT3*	Leucine-rich repeat transmembraneprotein FLRT3 precursor	Cell adhesion
Ssc.4664.1.S1_at	0.96	**1.04**	**1.16**	*PPP2R2C*	Serine/threonine protein phosphatase 2A	Signal transduction
Ssc.13849.1.S1_at	−0.92	**−1.04**	**−1.10**	*TINAGL1*	Androgen-regulated gene 1 [*H. sapiens*]	Immune response, polysaccharide binding
Ssc.7272.1.A1_at	−0.96	**−1.17**	**−1.14**	*SERPINB2*	Plasminogen activator inhibitor-2precursor	Protease inhibitor, anti-apoptosis
Ssc.9334.1.S1_at	−0.93	**−1.05**	**−1.35**	*RPIA*	Ribose-5-phosphate isomerase	Isomerase
Ssc.30734.1.S1_at	−0.64	**−1.01**	**−1.38**	*TSPAN7*	Transmembrane 4 superfamily member 2	Cell proliferation, cell motility
Ssc.3012.1.S1_at	−0.83	**−1.13**	**−1.46**	*UPP1*	Uridine phosphorylase 1	Glycosyltransferase, immune response
Ssc.9461.1.A1_at	−0.60	**−1.10**	**−1.85**	*ERRFI1*	Mitogen-inducible gene 6 protein	Response to stress, protein kinase binding
Ssc.2165.2.S1_a_at	−0.72	**−1.37**	**−1.86**	*SFN*	Stratifin; Epithelial cell marker protein 1	DNA damage response, apoptosis
Ssc.12463.1.A1_at	−0.97	**−1.74**	**−1.97**	*TRIM16*	Tripartite motif protein 16	Interleukin-1 binding
Ssc.24966.1.S1_at	−1.10	**−1.40**	**−2.47**	*NP*	Purine nucleoside phosphorylase	Glycosyltransferase, transferase
Ssc.15601.1.A1_s_at	−1.44	**−1.71**	**−2.65**	*IL1B*	Interleukin-1 beta precursor	Inflammatory response, cytokine, pyrogen
Ssc.29329.1.A1_at	−0.97	**−1.61**	**−2.71**	*DCHS2*	Protocadherin protein CDHJ [*H. sapiens*]	Cell adhesion, calcium ion binding
Ssc.17573.1.S1_at	−1.52	**−1.89**	**−2.72**	*IL1B*	Interleukin-1 beta precursor	Inflammatory response, cytokine, pyrogen
SscAffx.23.1.S1_at	−2.87	**−3.60**	**−3.68**	*IL17A*	Interleukin-17 precursor	Cytokine, inflammatory response

The selection criteria to define a transcript as differentially regulated (indicated in bold) were: an absolute log2 ratio equal or larger than 1, a MAS 5.0 present detection call and an uncorrected P value of <0.001. Transcripts not in bold are not differentially regulated as they do not meet these strict requirements.

Group IV (Table 10) includes genes exclusively found in one of the comparisons with themutant strains, eight genes specific for GIS26 (STa^−^ STb^low^ LT^−^) and two genes for GIS26 (STa^−^ STb^−^ LT^−^). However, these genes could not be related to ETEC infection and also don’t elucidate the influence of the different enterotoxins contributing.

**Table 10:** Microarray data expressed as a log2 fold-change of PBS and mutant ETEC-infected versus wild type ETEC-infected (WT) small intestinal segments at 4 h (Group IV, differentially regulated transcripts exclusively found in one of the mutant strain comparisons).

Probe Set ID	Log2 ratio			Genesymbol	Gene title	Tentative function
	GIS26 (STa^−^STb^low^ LT^−^)/WT	GIS26 (STa^−^STb^−^ LT^−^)/WT	PBS/WT			
Ssc.26516.1.A1_at	**1.00**	0.87	−0.84	*ABCG8*	ATP-binding cassette, sub-family G, member 8	Transport
Ssc.16332.1.S1_at	**1.01**	0.71	−0.90	*ABCC2*	Canalicular multispecific organic anion transporter 1	Transport
Ssc.17339.1.S1_at	**1.01**	0.81	−0.98	*SLC15A1*	Oligopeptide transporter, small intestineisoform (Intestinal H+/peptide cotransporter)	Digestion, protein transport
Ssc.5656.1.S1_at	**1.14**	0.87	−0.73	*TLL2*	Tolloid-like 2 [*H. sapiens*]	Developmental protein, hydrolase, protease, metalloprotease
Ssc.196.1.S1_at	**−1.55**	−1.21	1.49	*PLAT*	Tissue-type plasminogen activator precursor	Serine-type endopeptidase activity
Ssc.9311.1.A1_at	**−1.22**	−0.92	0.92	*PHLDA1*	Pleckstrin homology-like domain, family A, member 1 [*H. sapiens*]	Apoptosis, protein binding
Ssc.10552.1.A1_at	**−1.14**	−0.86	1.28	*PTPRG*	Protein-tyrosine phosphatase gammaprecursor	Hydrolase, protein phosphatase
Ssc.3139.1.A1_at	**−1.13**	−0.91	0.67	*RGS2*	Regulator of G-protein signaling 2	Signal transduction inhibitor
Ssc.11076.1.S1_at	−1.57	**−2.07**	1.93	*SDS*	L-serine dehydratase	Gluconeogenesis, lyase
Ssc.2464.1.S1_at	−1.35	**−1.80**	1.33	*STC1*	Stanniocalcin 1 precursor	Hormone activity, response to nutrient

The selection criteria to define a transcript as differentially regulated (indicated in bold) were: an absolute log2 ratio equal or larger than 1, a MAS 5.0 present detection call and an uncorrected P value of <0.001. Transcripts not in bold are not differentially regulated as they do not meet these strict requirements.

Group V (Table 11) represents genes found only in the PBS versus wild type strain comparison and not in the comparison of wild type with the mutant strains. These genes are most likely associated with LPS and/or metabolites of ETEC. They include immune related genes such as *PAP*, *MMP1*, *DUOX2* and *IL1RN*, and metabolism related genes as *MYO5B*, *SLC7A11*, *NP*, and *DCHS2*.

**Table 11:** Microarray data expressed as a log2 fold-change of PBS and mutant ETEC-infected versus wild type ETEC-infected (WT) small intestinal segments (Group V, differentially regulated transcripts only found in the comparison WT/PBS).

Probe Set ID	Log2 ratio			Genesymbol	Gene title	Tentative function
	GIS26 (STa^−^STb^low^ LT^−^)/WT	GIS26 (STa^−^STb^−^ LT^−^) /WT	PBS/WT			
Ssc.21194.1.S1_at	1.92	2.40	**3.46**	*PTPRR*	Receptor-type protein-tyrosine phosphatase R precursor	Hydrolase, protein phosphatase, receptor
Ssc.11487.1.A1_at	1.57	1.80	**2.66**	*PRR15*	–	Developmental protein
Ssc.18488.1.S1_at	1.69	1.64	**2.59**	*AQP8*	Aquaporin 8	Transport
Ssc.7458.1.A1_at	1.31	1.52	**2.15**	*GPT2*	Alanine aminotransferase 2 [*H. sapiens*]	Aminotransferase, transferase
Ssc.4724.1.S1_at	1.18	1.20	**2.14**	*PHLPPL*	–	Protein binding, catalytic activity
Ssc.16250.1.S2_at	−1.37	−1.63	**−2.01**	*IL1RN*	Interleukin-1 receptor antagonist protein precursor	Cytokine activity, interleukin-1 receptor antagonist activity, immune response, inflammatory response
Ssc.16013.1.S1_at	−1.00	−0.94	**−2.05**	*MMP1*	Interstitial collagenase precursor; matrix metalloproteinase-1	Proteolysis, metalloendopeptidase activity
Ssc.30857.1.S1_at	−0.80	−1.12	**−2.05**	*ENC1*	Ectoderm-neural cortex-1 protein	Ubl conjugation pathway
Ssc.19907.1.S1_at	−1.55	−1.55	**−2.22**	*F3*	Tissue factor precursor	Blood coagulation
Ssc.11609.1.A1_at	−1.10	−1.15	**−2.28**	*ASNS*	Asparagine synthetase	Ligase
Ssc.33.1.S1_at	−1.22	−0.99	**−2.33**	*DUOX2*	Dual oxidase 2 precursor[*H. sapiens*]	Oxidoreductase, peroxidase, cytokine-mediated signaling pathway
Ssc.29281.1.A1_at	−1.47	−1.35	**−2.35**	*SLC7A11*	Cystine/glutamate transporter	Response to toxin, transport
Ssc.18918.1.A1_at	−0.92	−1.01	**−2.43**	*GPX2*	Glutathione peroxidase-gastrointestinal	Oxidoreductase, peroxidase, response to oxidative stress
Ssc.6189.1.A1_at	−1.64	−1.52	**−2.90**	*SLC7A11*	Cystine/glutamate transporter	Response to toxin, transport
Ssc.16470.1.S1_at	−0.12	−0.48	**−3.10**	*REG3A (PAP)*	Pancreatitis-associated protein 1 precursor	Acute phase response, inflammatory response

The selection criteria to define a transcript as differentially regulated (indicated in bold) were: an absolute log2 ratio equal or larger than 2, a MAS 5.0 present detection call and an uncorrected P value of <0.001. Transcripts not in bold are not differentially regulated as they do not meet these strict requirements.

### Validation of the Microarray with qRT-PCR Analysis

Validation of expression differences measured with microarrays using an alternative method is essential [44]. This was done through quantifying the expression with RT-PCR on nine selected genes, eight differentially regulated immune response genes *IL1A*, *IL8*, *IL17A*, *PAP*, *TLR4*, *MMP1*, *MMP3*, *CYP1A1*, and a presumed constitutive reference gene, *FABP2* (Table 2). FABP2, also named intestinal fatty acid binding protein (I-FABP), is a specific marker for the relative amount of epithelium [45], and its constitutive expression should be unaffected by ETEC infection, which is the case here. No expression differences were found with qRT-PCR consistent with the microarray data.

**Figure 5:**
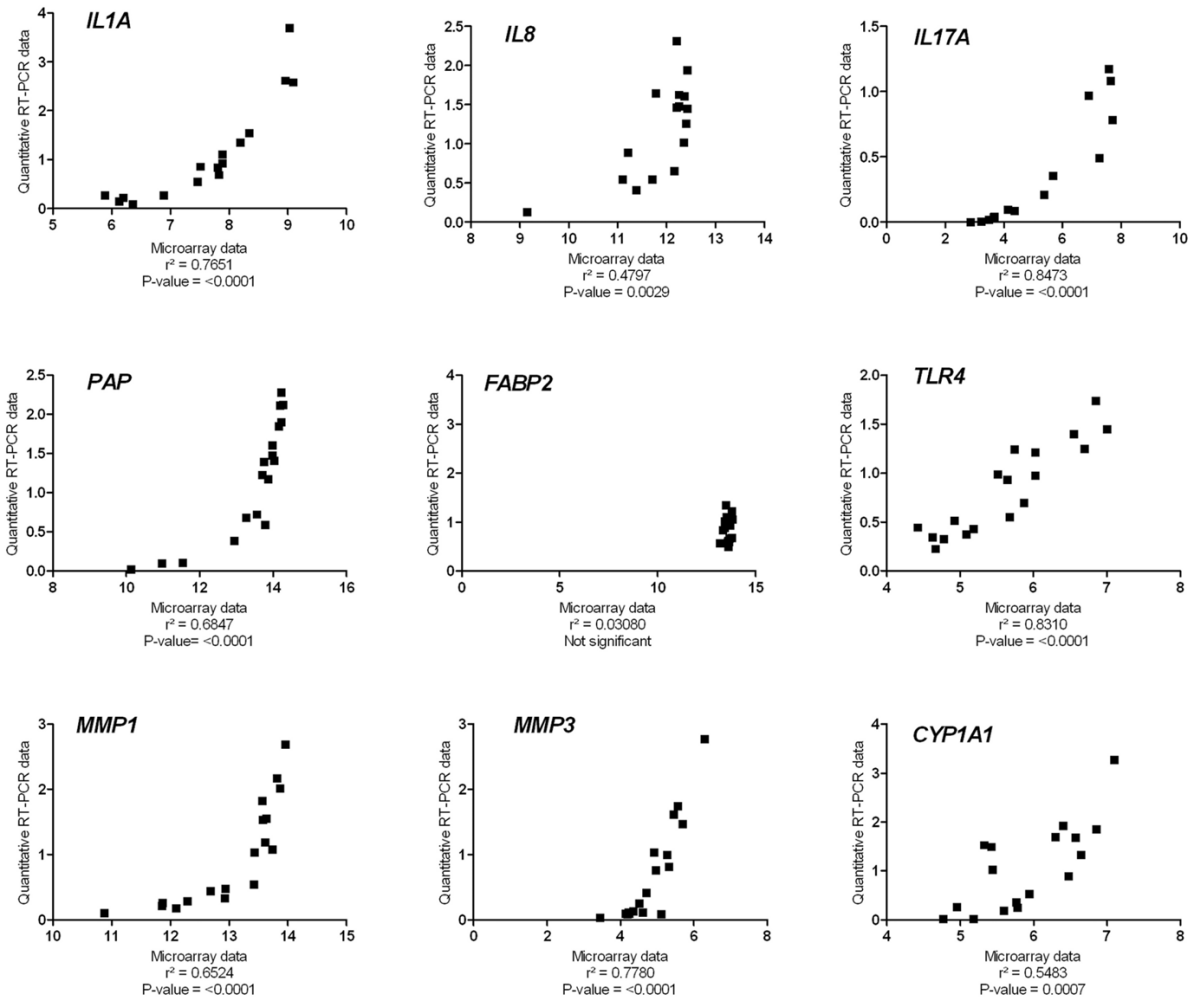
Linear regression of qRT-PCR CT ratios versus log2 expression ratios as obtained by microarray analysis for *IL1A*, *IL8*, *IL17A*, *PAP*, *FABP2*, *TLR4*, *MMP1*, *MMP3* and *CYP1A1*. The CT values for the genes of interest were normalized for two reference genes *RPL4*, and *GAPDH*. The ratios on the x- and y-axis were calculated as the log2 expression value of the experimental sample minus the log2 expression value of the control sample, for qRT-PCR data as well as microarray data. The microarray analysis was performed on pooled samples, and the qRT-PCR analysis on individual samples. The goodness of fit (r^2^) and *P*-value are given.

Linear regression analysis showed that the correlation between the values of the microarray and qRT-PCR data was highly significant for *IL1A*, *IL17A*, *PAP*, *TLR4*, *MMP3*, and *CYP1A1* and significant for *MMP1* and *IL8* (Figure 5).

## Discussion

The contributions of different enterotoxins of an F4^+^ ETEC strain to the induction of small intestinal secretion and early innate immune responses were studied in weaned piglets by use of isogenic deletion mutants. To our surprise, we were not able to obtain a mutant strain with an LT only phenotype. We have no direct explanation for the effect of deletion of one toxin gene (*eltAB* or *estA*) on the expression of other toxins. The methodology used is very gene specific and we always confirmed by PCR that only the target toxin gene was deleted. In addition, genome sequencing of the wild type GIS26 ETEC strain revealed that *eltAB* and *estA* are present on different virulence plasmids and therefore polar effects of the deletion of *eltAB* on *estA* and vice versa can be excluded. However, differences in toxin expression might be regulated at the level of transcription where one toxin controls the expression of another toxin but this requires further investigation. Therefore, due to the discrepancy between genotype and phenotype in some of the mutants, conclusions on toxin knock-out in the present study are based on the *in vitro* toxin phenotype characterization of the mutants. Although comparisons are not ideal, conclusions about the relative contribution of the different enterotoxins with respect to functionality and gene expression are still possible.

To compare the secretory effects of different bacterial strains within one piglet we used a small intestinal segment perfusion (SISP) technique. Results did not suggest an important role for LT or STa in the induction of secretion by the wild type GIS26 *E. coli* strain, since no significant difference was found between wild type and GIS26 (STa^−^ STb^+^ LT^−^) strains. Also, the relative unimportance of STa is further confirmed by the limited effect of GIS26 (STa^+^ STb^−^ LT^−^) on net absorption. Here, the age of the piglets could be of importance since neonatal animals are more susceptible to STa induced diarrhea [46]. However, results with GIS26 (STa^+^ STb^−^ LT^+^) also suggest that the combined effects of LT and STa on net absorption can lead to secretion in some pigs. This effect seems however variable and might be dependent on an underlying infection. One candidate is rotavirus. Several publications already suggested that diarrhea due to ETEC could be aggravated by a concurrent infection with rotavirus [47], [48], [49]. Previous studies with isogenic deletion mutants in a gnotobiotic infection model highlighted the importance of LT as a virulence factor compared to STb [13], [14], [15]. Our results suggest an important role for STb in the early secretory response. This difference could also be explained by a difference in age of the piglets used. Whereas previous studies used piglets less than two weeks old, piglets in our study were five weeks old. It has been described that presence of STb is more often associated with ETEC isolates from post-weaning diarrhea than from neonatal diarrhea [12], [50]. This could also explain why the number of isolates in which STb is present increases with the age of the animal [51]. For LT on the other hand, it has been shown *in vitro* that binding to its receptor GM_1_ on brush border vesicles is stronger in neonatal piglets compared to 4-week-old piglets [52]. Furthermore, the difference in sampling time and model used may also explain the conflicting results between this study and others. In the above mentioned studies [13], [14], [15] pigs were orally infected and clinical signs of diarrhea where recorded until 96 h after infection. Alternatively, 4 h could be too early for LT to note any appreciable effect. In a mouse intestinal loop model, secretory effects of STa and STb were already visible 30 minutes after administration, and any effect for LT on secretion was only noted 3 h after incubation with a maximal effect at 8 h [53]. Furthermore, unpublished studies of our lab could not demonstrate net fluid secretion via the intestinal segment perfusion technique the first 4 hours after incubation with 3 µg LT, whereas after 6 hours decreased absorption and sometimes secretion could be seen (unpublished data).

Having established that STb seems to be the most significant enterotoxin responsible for secretory responses, the correlation with gene expression was explored. The microarray analysis data were validated through the quantitative RT-PCR on eight selected immune genes and a reference gene (*FABP2*). A good correlation was obtained for the immune genes and for *FABP2* a constitutive expression was measured in both data sets.

First, a comparison was made between normal (PBS control) versus ETEC-infected small intestinal segments. The number of differentially expressed transcripts, 310 in total (38 transcripts when using an absolute log2-ratio larger than 2), was similar to an earlier study examining the influence of ETEC on gene expression, also using the SISP technique [54], paralleling the drastic change in fluid absorption. As expected, genes with a function in transport of fluids and electrolytes, such as *KCNJ13*, *AQP8*, *ATG10*, and *APOC3*, were significant differential down-regulated in ETEC infected segments. The apical chloride anion exchanger DRA (*SLC26A3*) is functionally coupled to CFTR (cystic fibrosis transmembrane regulator) and NHE3 (Na/H exchanger-isoform 3), which are both involved in the secretory pathways of LT, STa and STb [34], [35]. The transcript for this gene was significantly up-regulated in ETEC infected segments, clearly demonstrating its involvement in the disturbance of water and electrolyte transport after ETEC infection.

The physiological response to ETEC is also accompanied by a marked change in mucosal expression of innate immune genes. From the 38 transcripts (absolute log2-ratio larger than 2), 15 genes including *PAP* and *MMP1* appeared to be associated with ETEC infection irrespective of the enterotoxins produced (Table 11, group V). Niewold *et al*. [54] already suggested a possible role for PAP and MMP1 in ETEC infection, and they were also found in reaction to *Salmonella typhimurium* [55] and *Lactobacillus plantarum* [56], suggesting them to be important in a general antibacterial response. This is probably consistent with the established function for PAP as serum marker for Crohn’s disease [40], which may be also applicable for ETEC infection. IL-8 was found in the same general response group as PAP en MMP1, but with a lower expression level (absolute log2-ratio between 1 and 2) (Table S1). Its induction by ETEC may be in agreement with its apparent important role in infection resolution of ETEC [20]. Indeed, when piglets are infected with the F4^+^ GIS26 (WT) strain it results in a rapid colonization and a fast F4 specific mucosal immune response [57]. *In vitro* results, with the same ETEC strain, indicated that flagellin is involved in the induction of IL-8 [58], regardless of F4. This is in agreement with the absence of a differential regulation of IL-8 in our mutant strains, being all flagellin positive.

Further comparisons were done to establish gene expression associated with specific enterotoxins produced by ETEC. The comparisons GIS26 (STa^−^ STb^+^ LT^−^) versus wild type and GIS26 (STa^+^ STb^−^ LT^−^) versus WT showed no differential expression, showing that the presence of LT had no influence on the early gene expression following ETEC infection and indicating that presence of either one of the heat stable enterotoxins is sufficient to activate the early immune responses. Comparison GIS26 (STa^−^ STb^low^ LT^−^) versus WT showed the difference in gene expression (43 transcripts) due to the 3-fold lower STb concentration as in comparison GIS26 (STa^−^ STb^+^ LT^−^) versus WT (Figure 2B). Comparison GIS26 (STa^−^ STb^−^ LT^−^) versus GIS26 (STa^+^ STb^+^ LT^+^) showed 54 transcripts associated with presence or absence of all three toxins. Subsequently, transcripts were grouped according to genes in common between the three comparisons in which differential expression was found. This is not necessarily a functional grouping, and in fact only groups I and V could be related to specific factors. Group I represents genes related to STb, group V represents genes unrelated to enterotoxins (see above). The other groups cannot be easily related to specific factors, however, they allow for comparison between strains.

From the long list (Tables 7, 8, 9, 10, 11 and Table S1), only the most prominent genes and those earlier implicated in secretory bacterial pathogenesis are discussed in this paper. The matrix-metalloproteinase, MMP3, reported as critical for CD4^+^ T lymphocyte migration in the intestinal mucosa [59], was significantly up-regulated in the wild type strain. A previous study on acute cholera also demonstrated the expression of matrix metalloproteinases (MMP1 and MMP3) in duodenal mucosa [60]. Whereas MMP1 seems to be part of the general antibacterial response (see above), MMP3 may be specific for Gram-negative bacteria that cause severe secretory diarrhea. Furthermore, our results with the mutant strains suggest that *MMP3*, as all genes of group I (Table 7), is at least partially regulated by the heat stable enterotoxins expressed. This is in agreement with previous studies which have shown an increase in prostaglandin E_2_ (PGE_2_) synthesis by STb *in vivo* [34]. MMP production has been shown to be PGE_2_-regulated in various cell types [61], [62], [63], [64]. We therefore speculate that high levels of STb are important in the induction of these immune genes and that STa might be able to compensate for the loss of STb.

The genes *SERPINE1* and *TLR4*, both involved in immune/inflammatory responses, are inducible by lipopolysaccharide, present in the outer layer of Gram-negative bacteria [65], [66], [67], [68]. For the porcine TLR4 gene this has also been confirmed in LPS-stimulated porcine dendritic cells and an intestinal epithelial cell line [69], [70]. In addition *Vibrio cholerae*, secreting cholera toxin (an enterotoxin homologous to LT), induced TLR4 expression *in vitro* in a human IEC [71]. Our results showed no differential regulation of these genes in segments infected with the Gram-negative GIS26 ETEC strain (comparison 3, Table 8). For TLR4, this is in agreement with an *in vitro* study on porcine epithelial cells where an STa secreting ETEC strain even seemed to down-regulate TLR4 expression at very high concentrations [69]. A previous microarray study on the porcine intestinal epithelial cell line IPEC-J2 also lacked induction of TLR4 after co-incubation with a LT^+^ STb^+^ F4ac ETEC strain, compared to mock-infected cells [32]. A down-regulated expression of TLR4 in the segments infected with mutant strains GIS26 (STa^−^ STb^low^ LT^−^) and GIS26 (STa^−^ STb^−^ LT^−^) was found here. Taken together, it is suggested that *TLR4* and *SERPINE1* are not solely regulated by LPS from ETEC but are rather down-regulated in the absence of LT, STa and (most of) STb.

In group III of Table 9, strongly regulated immune genes are *IL1B* and *IL17A*. IL-17 is generally thought to increase inflammation by recruiting other immune cells. CD4^+^ Th17 cells, characterized by the production of IL-17 [72], are probably involved in clearance of extracellular pathogens [73], [74], [75]. They have also been shown to play an important role in the pathogenesis of colitis and several other autoimmune diseases (reviewed in [76], [77]). Furthermore, much of the IL-17 released during an inflammatory response is produced by innate immune cells including granulocytes and monocytes [78]. These early responses have a central role in the initiation of IL-17-dependent immune responses, even before the activation of Th17 cells (reviewed in [79]). Here, *IL17A* was found to be upregulated by ETEC (PBS versus WT comparison), and the GIS26 (STa^−^ STb^−^ LT^−^) mutant lacked this upregulation (GIS26 (STa^−^ STb^−^ LT^−^) versus WT comparison) (Table 7). This suggests the *IL17A* response to be enterotoxin specific. Since LT does not seem to have an influence on gene expression (Table 4), it is suggested that STa or STb are responsible. From the comparisons GIS26 (STa^−^ STb^low^ LT^−^) and GIS26 (STa^−^ STb^−^ LT^−^) versus the wild type strain, it can be concluded that already limited amounts of STb (STb^low^), are sufficient to elicit an *IL17A* response. A similar reaction is seen with *IL1B*. Since there is no difference in gene expression between the wild type strain and GIS26 (STa^+^ STb^−^ LT^−^), presence of only STa also seems sufficient to induce these responses.

In summary, our data suggest that the wild type ETEC strain used in this study can influence immune responses by a variety of pathways. Results from this study can be useful to select either targets for intervention or parameters to measure severity of intestinal diseases. This is also the first study to investigate both the functional role of ETEC enterotoxins and their possible influence on ETEC induced innate immune responses. Our data show the existence of at least two different responses; first what appears to be a general antibacterial response, comprising genes such as *PAP*, *MMP1* and *IL8* and second, a heat-stable enterotoxin specific response, comprising genes such as *IL17A* and *IL1B*.

## Supporting Information

Table S1 **Microarray data expressed as a log2 fold change of PBS and mutant ETEC-infected verus wild type-infected (WT) small intestinal segments.**
(DOCX)
