# Supplementary material for: Role of Heat-Stable Enterotoxins in the Induction of Early Immune Responses in Piglets after Infection with Enterotoxigenic Escherichia coli
Source: PLoS One. 2012 Jul 17;7(7):e41041. doi: 10.1371/journal.pone.0041041 (PMC3398878; doi:10.1371/journal.pone.0041041)
Supplement: Table S1 — Microarray data expressed as a log2 fold change of PBS and mutant ETEC-infected verus wild type-infected (WT) small intestinal segments. (DOCX) [file pone.0041041.s001.docx]

| **Probe Set ID** | **Log2 ratio** | | | | **Gene symbol** | **P value** | | | **Gene title** | **Tentative function** |
| --- | --- | --- | --- | --- | --- | --- | --- | --- | --- | --- |
|  | **GIS26**  **(STa^‑^ STb^low^ LT ^‑^) /WT** | | **GIS26 (STa^‑^STb^‑^ LT ^‑^) /WT** | **PBS /WT** |  | **GIS26**  **(STa^‑^ STb^low^ LT ^‑^) /WT** | **GIS26 (STa^‑^STb^‑^ LT ^‑^) /WT** | **PBS /WT** |  |  |
| Ssc.22959.1.S1_at | 2,83 | | 3,24 | 3,96 | *PCK1* | 5,48E-04 | 1,64E-04 | 2,26E-05 | Phosphoenolpyruvate carboxykinase, cytosolic [GTP] | Gluconeogenesis |
| Ssc.29525.1.A1_at | 1,62 | | 1,88 | 2,37 | *ATG10* | 7,74E-04 | 2,12E-04 | 2,10E-05 | APG10 autophagy 10-like *[H. sapiens]* | Ligase, autophagy, protein transport, transport, Ubl conjugation pathway |
| Ssc.16864.1.S1_at | 1,42 | | 1,44 | 2,37 | *PPARGC1A* | 6,98E-04 | 6,16E-04 | 4,62E-06 | Peroxisome proliferator activated receptor gamma coactivator 1 alpha (PPAR gamma coactivator-1 alpha) | Transcription, transcription activator |
| Ssc.7301.1.A1_at | 1,75 | | 1,81 | 2,22 | *CDC10* | 2,37E-06 | 1,65E-06 | 1,43E-07 | Septin 7 | Cytokinesis, mitosis, cell cycle |
| Ssc.5000.1.A1_at | 1,23 | | 1,32 | 1,85 | *ERBB2* | 3,23E-04 | 1,79E-04 | 5,50E-06 | Receptor protein-tyrosine kinase erbB-2 precursor | Activator, kinase, receptor, transferase, tyrosine-protein kinase |
| Ssc.298.1.S1_at | 1,78 | | 1,53 | 1,85 | *PRSS7* | 1,06E-04 | 4,21E-04 | 7,33E-05 | Enteropeptidase precursor | Hydrolase, protease, serine protease |
| Ssc.10602.1.A1_at | 1,08 | | 1,15 | 1,59 | *FLRT3* | 2,22E-05 | 1,14E-05 | 2,70E-07 | Leucine-rich repeat transmembrane protein FLRT3 precursor | Cell adhesion |
| Ssc.20832.1.S1_at | 1,26 | | 1,24 | 1,55 | *SCTR* | 1,31E-04 | 1,58E-04 | 1,70E-05 | Secretin receptor precursor | G-protein coupled receptor, receptor, transducer |
| Ssc.18915.1.A1_at | 1,23 | | 1,30 | 1,54 | *ZC3H11A* | 8,22E-04 | 4,98E-04 | 1,08E-04 | Zinc finger CCCH domain-containing protein 11A | Nucleic acid-, zinc ion-, protein binding |
| Ssc.14573.1.S1_at | 1,01 | | 1,08 | 1,77 | *EYA2* | 5,93E-04 | 3,21E-04 | 2,10E-06 | Eyes absent homolog 2 | Activator, chromatin regulator, developmental protein, hydrolase, protein phosphatase, transcription regulation |
| Ssc.27422.1.A1_at | 1,19 | | 1,19 | 1,52 | *ACBD5* | 3,27E-04 | 3,47E-04 | 3,24E-05 | acyl-Coenzyme A binding domain containing 5 *[H. sapiens]* | Transport |
| Ssc.17849.1.A1_at | 1,44 | | 1,20 | 1,51 | *SLC30A10* | 7,98E-05 | 4,49E-04 | 4,90E-05 | Solute carrier family 30; zinc transporter 8 *[H. sapiens]* | Ion tranport, transport, zinc transport |
| Ssc.208.1.S1_at | 1,32 | | 1,30 | 1,46 | *CYP1A1* | 4,34E-04 | 4,94E-04 | 1,77E-04 | Cytochrome P450 1A1 | Monooxygenase, oxidoreductase |
| Ssc.7116.1.A1_at | 1,18 | | 1,25 | 1,39 | *NT5C3* | 5,65E-04 | 3,47E-04 | 1,36E-04 | 5-nucleotidase, cytosolic III  *[H. sapiens]* | Hydrolase, transferase |
| Ssc.10703.1.A1_at | 1,52 | | 1,40 | 1,02 | *SLC25A27* | 5,79E-03 | 4,47E-02 | 9,74E-05 | Mitochondrial uncoupling protein 4 (UCP 4) | Transport |
| Ssc.26709.1.S1_at | -1,11 | | -1,22 | -1,12 | *GPR183* | 8,65E-04 | 3,75E-04 | 8,02E-04 | EBV-induced G protein-coupled receptor 2 (EBI2) | Adaptive immunity, immunity, humoral immune response |
| Ssc.3509.1.S1_at | -1,18 | | -1,14 | -1,30 | *HK2* | 5,54E-05 | 7,66E-05 | 2,05E-05 | Hexokinase, type II | Kinase, transferase |
| Ssc.11194.1.S1_at | -1,18 | | -1,28 | -1,32 | *PLAU* | 2,06E-04 | 9,44E-05 | 7,18E-05 | Urokinase-type plasminogen activator precursor | Blood coagulationn fibrinolysis, plasminogen activation |
| Ssc.18603.1.A1_at | -1,57 | | -1,80 | -2,07 | *G0S2* | 7,13E-04 | 2,22E-04 | 5,81E-05 | Putative lymphocyte G0/G1 switch protein 2 | Cell cycle |
| Ssc.12431.1.A1_at | -2,40 | | -2,46 | -2,11 | *MYO5B* | 2,86E-04 | 2,23E-04 | 8,43E-04 | Myosin Vb | Protein transport |
| Ssc.113.1.S1_at | -1,79 | | -1,77 | -2,30 | *IL1A* | 7,19E-04 | 7,93E-04 | 7,51E-05 | Interleukin-1 alpha precursor | Inflammatory response, cytokine, pyrogen |
| Ssc.30277.1.A1_at | -1,79 | | -1,88 | -2,46 | *SLC26A3* | 6,93E-04 | 4,42E-04 | 3,58E-05 | Chloride anion exchanger | Antiport, transport (excretion) |
| Ssc.113.1.S2_at | -1,69 | | -1,82 | -2,68 | *IL1A* | 5,29E-04 | 2,69E-04 | 5,46E-06 | Interleukin-1 alpha precursor | Inflammatory response, cytokine, pyrogen |
| Ssc.15927.1.S1_at | -2,30 | | -2,28 | -4,18 | *MMP3* | 6,85E-04 | 7,24E-04 | 1,65E-06 | Stromelysin-1 precursor; Matrix metalloproteinase-3 | Proteolysis, metalloendopeptidase activity |
| Ssc.7991.1.A1_at | 1,84 | | 1,47 | 2,16 | *KIAA1468* | 3,23E-04 | 2,08E-03 | 7,28E-05 | KIAA1468 | Binding |
| Ssc.1039.1.S1_at | 1,74 | | 1,47 | 2,00 | *APOC3* | 2,64E-04 | 1,10E-03 | 7,17E-05 | Apolipoprotein C-III precursor | Transport, G-protein coupled receptor protein signaling pathway |
| Ssc.24638.1.S1_at | 1,42 | | 1,27 | 1,67 | *PRLR* | 9,22E-04 | 2,27E-03 | 2,33E-04 | Prolactin receptor precursor | Receptor, T cell activation |
| Ssc.1147.1.A1_at | -1,18 | | -0,95 | -1,76 | *LPL* | 4,97E-05 | 3,80E-04 | 6,05E-07 | Lipoprotein lipase precursor | Lipid degradation, hydrolase |
| Ssc.15927.2.A1_at | -1,72 | | -1,58 | -2,90 | *MMP3* | 8,07E-04 | 1,58E-03 | 4,83E-06 | Stromelysin-1 precursor; Matrix metalloproteinase-3 | Proteolysis, metalloendopeptidase activity |
| Ssc.15927.2.S1_at | -2,28 | | -2,23 | -4,16 | *MMP3* | 9,31E-04 | 1,13E-03 | 2,53E-06 | Stromelysin-1 precursor; Matrix metalloproteinase-3 | Proteolysis, metalloendopeptidase activity |
| Ssc.18284.1.A1_at | 2,45 | | 2,86 | 3,41 | *KCNJ13* | 1,12E-03 | 3,11E-04 | 5,92E-05 | Inward rectifier potassium channel 13 | Voltage-gated channel, ion transport, potassium transport, transport |
| Ssc.9238.1.A1_at | 1,08 | | 1,64 | 2,27 | *ATP10D* | 4,37E-03 | 1,47E-04 | 4,94E-06 | Potential phospholipid-transporting ATPase VD | Hydrolase |
| Ssc.20419.2.S1_at | 1,11 | | 1,86 | 2,02 | *SLC25A27* | 3,57E-03 | 6,16E-04 | 1,85E-05 | Mitochondrial uncoupling protein 4 (UCP 4) (Solute carrier family 25, member 27) | Transport |
| Ssc.22210.2.S1_at | 1,34 | | 1,45 | 1,99 | *MTHFD2L* | 1,63E-03 | 8,77E-04 | 4,91E-05 | similar to Bifunctional methylenetetrahydrofolate dehydrogenase/cyclohydrolase *[H. sapiens]* | Hydrolase, oxidoreductase |
| Ssc.27342.1.S1_at | 1,11 | | 1,34 | 1,85 | *ONECUT2* | 3,81E-03 | 9,45E-04 | 5,10E-05 | - | Transcriptional activator |
| Ssc.27502.1.S1_at | 1,38 | | 1,73 | 1,85 | *ITGB8* | 1,56E-03 | 2,41E-04 | 1,27E-04 | Intergrin beta-8 | Integrin, receptor, cell adhesion |
| Ssc.24037.1.S1_at | 1,14 | | 1,44 | 1,83 | *UBE2U* | 2,14E-03 | 3,06E-04 | 3,03E-05 | - | Ubl conjugation pathway |
| Ssc.28515.1.S1_at | 1,13 | | 1,25 | 1,61 | *USP2* | 1,09E-03 | 4,66E-04 | 4,29E-05 | Ubiquitin carboxyl-terminal hydrolase 2 | Cell cycle, myogenesis, Ubl conjugation pathway |
| Ssc.2132.1.S1_a_at | 1,03 | | 1,16 | 1,53 | *RPS6KA5* | 1,15E-03 | 4,14E-04 | 2,92E-05 | Ribosomal protein S6 kinase alpha 5 | Kinase, serine/threonine-protein kinase, transferase, response to stress and external stimulus |
| Ssc.30861.1.A1_at | 0,93 | | 1,11 | 1,33 | *FLRT3* | 3,91E-04 | 7,04E-05 | 1,16E-05 | Leucine-rich repeat transmembrane protein FLRT3 precursor | Cell adhesion |
| Ssc.4664.1.S1_at | 0,96 | | 1,04 | 1,16 | *PPP2R2C* | 1,60E-04 | 7,40E-05 | 2,41E-05 | Serine/threonine protein phosphatase 2A, 55 kDa regulatory subunit B, gamma isoform | Signal transduction |
| Ssc.13849.1.S1_at | -0,92 | | -1,04 | -1,10 | *TINAGL1* | 1,20E-03 | 4,58E-04 | 2,73E-04 | P3ECSL; androgen-regulated gene 1 *[H. sapiens]* | Immune response, polysaccharide binding |
| Ssc.7272.1.A1_at | -0,96 | | -1,17 | -1,14 | *SERPINB2* | 5,27E-04 | 8,70E-05 | 1,08E-04 | Plasminogen activator inhibitor-2 precursor (PAI-2) | Protease inhibitor, serine protease inhibitor, anti-apoptosis |
| Ssc.9334.1.S1_at | -0,93 | | -1,05 | -1,35 | *RPIA* | 4,46E-04 | 1,48E-04 | 1,19E-05 | Ribose-5-phosphate isomerase | Isomerase |
| Ssc.30734.1.S1_at | -0,64 | | -1,01 | -1,38 | *TSPAN7* | 6,59E-03 | 1,98E-04 | 8,68E-06 | Transmembrane 4 superfamily member 2 | Cell proliferation, cell motility |
| Ssc.3012.1.S1_at | -0,83 | | -1,13 | -1,46 | *UPP1* | 7,35E-03 | 7,58E-04 | 7,82E-05 | Uridine phosphorylase 1 | Glycosyltransferase, transferase, immune response |
| Ssc.9461.1.A1_at | -0,60 | | -1,10 | -1,85 | *ERRFI1* | 1,52E-02 | 1,76E-04 | 7,01E-07 | Mitogen-inducible gene 6 protein | Response to stress, protein kinase binding |
| Ssc.2165.2.S1_a_at | -0,72 | | -1,37 | -1,86 | *SFN* | 6,01E-03 | 2,49E-05 | 8,58E-07 | 14-3-3 protein sigma (Epithelial cell marker protein 1) | DNA damage response, signal transduction resulting in induction of apoptosis |
| Ssc.12463.1.A1_at | -0,97 | | -1,74 | -1,97 | *TRIM16* | 9,03E-03 | 8,91E-05 | 2,56E-05 | Tripartite motif protein 16 | Interleukin-1 binding |
| Ssc.24966.1.S1_at | -1,10 | | -1,40 | -2,47 | *NP* | 3,23E-03 | 4,73E-04 | 1,41E-06 | Purine nucleoside phosphorylase | Glycosyltransferase, transferase |
| Ssc.15601.1.A1_s_at | -1,44 | | -1,71 | -2,65 | *IL1B* | 2,20E-03 | 5,37E-04 | 7,50E-06 | Interleukin-1 beta precursor | Inflammatory response, cytokine, pyrogen |
| Ssc.29329.1.A1_at | -0,97 | | -1,61 | -2,71 | *DCHS2* | 3,72E-03 | 4,63E-05 | 1,33E-07 | Dachsous 2 isoform 1; protocadherin protein PCDHJ *[H. sapiens]* | Cell adhesion, calcium ion binding |
| Ssc.17573.1.S1_at | -1,52 | | -1,89 | -2,72 | *IL1B* | 1,57E-03 | 2,45E-04 | 6,20E-06 | Interleukin-1 beta precursor | Cytokine, inflammatory response |
| SscAffx.23.1.S1_at | -2,87 | | -3,60 | -3,68 | *IL17A* | 1,27E-03 | 1,82E-04 | 1,49E-04 | Interleukin-17 precursor; Cytotoxic T lymphocyte-associated antigen 8 | Cytokine, inflammatory response |
| Ssc.9781.1.S1_at | -1,36 | | -1,25 | -1,11 | *SERPINE1* | 2,44E-04 | 5,15E-04 | 1,36E-03 | Plasminogen activator inhibitor-1 precursor | Plasminogen activation, cellular response to lipopolysaccharide, defense response to Gram-negative bacterium, positive regulation of IL-8 production, positive regulation of leukotriene production involved in inflammatory response |
| Ssc.12781.1.A1_at | -1,16 | | -1,26 | -0,94 | *TLR4* | 7,20E-04 | 3,45E-04 | 3,49E-03 | Toll-like receptor 4 precursor | Immunity, inflammatory response, innate immunity, lipopolysaccharide receptor activity |
| Ssc.1674.1.A1_at | -1,09 | | -1,08 | -0,74 | *SLC2A14* | 6,34E-04 | 6,95E-04 | 9,76E-03 | Glucose transporter 14 *[H. sapiens]* | Developmental protein |
| Ssc.21194.1.S1_at | 1,92 | | 2,40 | 3,46 | *PTPRR* | 9,40E-03 | 2,10E-03 | 8,97E-05 | Receptor-type protein-tyrosine phosphatase R precursor | Hydrolase, protein phosphatase, receptor |
| Ssc.20419.1.S1_at | 1,52 | | 1,86 | 2,71 | *SLC25A27* | 8,27E-04 | 1,42E-04 | 2,77E-06 | Mitochondrial uncoupling protein 4: Solute carrier family 25, member 27 | Oxidoreductase |
| Ssc.11487.1.A1_at | 1,57 | | 1,80 | 2,66 | *PRR15* | 3,00E-03 | 1,08E-03 | 3,03E-05 | Proline-rich protein 15 | Developmental protein |
| Ssc.18488.1.S1_at | 1,69 | | 1,64 | 2,59 | *AQP8* | 3,15E-03 | 3,96E-03 | 8,61E-05 | Aquaporin 8 | Transport |
| Ssc.7458.1.A1_at | 1,31 | | 1,52 | 2,15 | *GPT2* | 1,42E-02 | 5,99E-03 | 4,23E-04 | Alanine aminotransferase 2; glutamic-pyruvate transaminase 2 *[H. sapiens]* | Aminotransferase, transferase |
| Ssc.4724.1.S1_at | 1,18 | | 1,20 | 2,14 | *PHLPPL* | 2,48E-03 | 2,18E-03 | 1,05E-05 | PHLPPL protein | Protein binding, catalytic activity |
| Ssc.22914.1.A1_at | 0,98 | | 1,07 | 1,97 | *PPARGC1A* | 1,79E-02 | 1,11E-02 | 9,91E-05 | Peroxisome proliferator activated receptor gamma coactivator 1 alpha | Transcription, transcription activator |
| Ssc.20570.1.S1_at | | 1,09 | 1,11 | 1,94 | *ACAD11* | 1,12E-02 | 9,92E-03 | 1,42E-04 | Nephrocystin 3 *[H. sapiens]* | Oxidoreductase |
| Ssc.22210.1.A1_at | | 1,22 | 1,29 | 1,84 | *MTHFD2L* | 1,97E-03 | 1,27E-03 | 5,25E-05 | Similar to Bifunctional methylenetetrahydrofolate dehydrogenase/cyclohydrolase *[H. sapiens]* | Hydrolase, oxidoreductase |
| Ssc.9684.1.S1_at | | 1,05 | 0,95 | 1,74 | *SLC16A10* | 7,06E-03 | 1,27E-02 | 1,33E-04 | Solute carrier family 16, member 10; T-type amino acid transporter 1 *[H. sapiens]* | - |
| Ssc.24797.1.A1_at | | 0,60 | 1,15 | 1,63 | *VTI1A* | 1,08E-01 | 5,36E-03 | 3,64E-04 | Vesicle transport through interaction with t-SNAREs homolog 1A | Protein transport, transport |
| Ssc.6646.1.S1_at | | 0,71 | 1,03 | 1,61 | *GRB7* | 1,62E-02 | 1,37E-03 | 2,38E-05 | Growth factor receptor-bound protein 7 | Epidermal growth factor receptor signaling pathway |
| Ssc.26633.1.A1_at | | 0,97 | 0,94 | 1,57 | *SLC16A10* | 4,70E-03 | 5,56E-03 | 8,77E-05 | Solute carrier family 16, member 10; T-type amino acid transporter 1  *[H. sapiens]* | Transport |
| Ssc.29970.1.A1_at | | 0,98 | 1,18 | 1,56 | *HTR2C* | 7,96E-03 | 2,39E-03 | 2,30E-04 | 5-hydroxytryptamine 2C receptor | G-protein coupled receptor, receptor, transducer |
| Ssc.16538.1.S1_at | | 1,37 | 1,21 | 1,55 | *C1orf168* | 7,92E-05 | 2,47E-04 | 2,24E-05 | - | - |
| Ssc.30512.1.A1_at | | 1,07 | 1,16 | 1,48 | *CA7* | 6,25E-03 | 3,80E-03 | 5,88E-04 | Carbonic anhydrase VII | Lyase |
| Ssc.29623.1.A1_at | | 0,25 | 0,61 | 1,47 | *RNF144A* | 4,79E-01 | 1,01E-01 | 8,77E-04 | Ubiquitin conjugating enzyme 7 interacting protein 4 | Ubl conjugation pathway |
| Ssc.4729.1.S2_at | | 0,64 | 0,71 | 1,44 | *Q7Z2T0* | 5,16E-02 | 3,23E-02 | 2,83E-04 | MSTP111 | - |
| Ssc.7686.1.S1_at | | 0,91 | 0,95 | 1,44 | *ATRN* | 6,36E-03 | 5,03E-03 | 1,83E-04 | Attractin precursor (Mahogany homolog) (DPPT-L) | Inflammatory response, receptor |
| Ssc.24762.1.A1_at | | 0,96 | 0,89 | 1,43 | *OSBPL1A* | 4,62E-03 | 7,33E-03 | 1,93E-04 | Oxysterol binding protein-related protein 1 | Lipid transport, transport |
| Ssc.22731.1.S1_at | | 1,04 | 0,87 | 1,41 | *SEC14L2* | 4,57E-03 | 1,35E-02 | 4,40E-04 | SEC14-like protein 2 | Transcription ,transport, transcription regulation |
| Ssc.4895.1.S1_at | | 0,93 | 0,94 | 1,41 | *FBXO25* | 4,07E-03 | 3,80E-03 | 1,32E-04 | F-box only protein 25 | Ubl conjugation pathway |
| Ssc.24543.1.S1_at | | 0,81 | 0,85 | 1,41 | *AFF3* | 3,86E-04 | 2,37E-04 | 1,22E-06 | LAF-4 protein (Lymphoid nuclear protein related to AF4) | Transcription, transcription regulation |
| Ssc.10131.1.A1_at | | 0,66 | 1,23 | 1,40 | *PDK4* | 6,88E-02 | 2,36E-03 | 9,05E-04 | Pyruvate dehydrogenase [lipoamide] kinase isozyme 4, mitochondrial precursor | Carbohydrate metabolism, glucose metabolism |
| Ssc.12497.1.A1_at | | 0,87 | 0,76 | 1,39 | *GAL3ST1* | 1,39E-03 | 3,85E-03 | 1,90E-05 | Galactosylceramide sulfotransferase | Transferase |
| Ssc.10444.1.S1_at | | 0,70 | 0,78 | 1,38 | *IL33* | 2,85E-02 | 1,68E-02 | 2,95E-04 | Interleukin-33 | Cytokine |
| Ssc.31120.1.A1_at | | 0,91 | 0,97 | 1,36 | *NCOA1* | 1,07E-03 | 6,43E-04 | 2,49E-05 | Nuclear receptor coactivator 1 isoform 1 *[H. sapiens]* | Transcription, transcription regulation |
| Ssc.16926.1.S1_at | | 1,14 | 0,97 | 1,36 | *SLC2A5* | 1,20E-03 | 4,00E-03 | 2,72E-04 | Solute carrier family 2, facilitated glucose transporter, member 5 (Glucose transporter type 5, small intestine) | Sugar transport, transport |
| Ssc.2697.1.S1_at | | 0,91 | 0,79 | 1,32 | *ADCY10* | 4,25E-03 | 1,04E-02 | 2,21E-04 | Soluble adenylyl cyclase *[H. sapiens]* | Lyase |
| Ssc.22660.1.S1_at | | 0,81 | 0,90 | 1,30 | *C9orf5* | 4,71E-03 | 2,18E-03 | 9,72E-05 | Transmembrane protein | Integral to membrane |
| Ssc.26321.1.S1_s_at | | 1,12 | 0,81 | 1,29 | *CYP2C18* | 2,35E-03 | 1,78E-02 | 7,60E-04 | Cytochrome P450 2C18 | Monooxygenase, oxidoreductase |
| Ssc.2274.1.A1_at | | 0,44 | 0,64 | 1,29 | *EFNA1* | 1,39E-01 | 3,94E-02 | 4,18E-04 | Ephrin-A1 precursor; Immediate early response protein B61; Tumor necrosis factor, alpha-induced protein 4 | Angiogenesis |
| Ssc.18435.1.A1_at | | 0,95 | 0,80 | 1,29 | *TMEM139* | 3,06E-04 | 1,27E-03 | 1,46E-05 | - | Integral to membrane |
| Ssc.4895.2.S1_at | | 0,97 | 0,91 | 1,28 | *FBXO25* | 6,36E-03 | 9,06E-03 | 8,18E-04 | F-box only protein 25 | Ubl conjugation pathway |
| Ssc.19637.1.S1_at | | 0,75 | 0,56 | 1,28 | *IL34* | 9,86E-05 | 1,20E-03 | 2,66E-07 | Interleukin-34 | Cytokine, growth factor |
| Ssc.30404.1.A1_at | | 0,67 | 0,83 | 1,28 | *KIAA0586* | 1,67E-03 | 2,86E-04 | 3,59E-06 | Uncharacterized protein KIAA0586 | - |
| Ssc.8609.1.A1_at | | 0,80 | 0,92 | 1,26 | *PDCD4* | 5,49E-04 | 1,54E-04 | 5,97E-06 | Programmed cell death 4 isoform 1; nuclear antigen H731 *[H. sapiens]* | Apoptosis |
| Ssc.3112.2.S1_at | | 0,98 | 0,75 | 1,25 | *COBL* | 2,51E-03 | 1,41E-02 | 3,72E-04 | Cordon-bleu homolog *[H. sapiens]* | - |
| Ssc.6106.1.S1_at | | 0,83 | 0,78 | 1,24 | *PTPRD* | 2,59E-03 | 4,01E-03 | 8,58E-05 | Protein-tyrosine phosphatase delta precursor | Hydrolase, protein phosphatase, receptor |
| Ssc.19163.1.S1_at | | 0,58 | 0,65 | 1,23 | *MXI1* | 4,17E-03 | 1,95E-03 | 4,98E-06 | MAX interacting protein 1 | Transcription, transcription regulation, repressor |
| Ssc.13060.1.S1_a_at | | 0,83 | 0,79 | 1,22 | *PAPD5* | 8,67E-05 | 1,47E-04 | 1,41E-06 | PAP associated domain containing 5 *[H. sapiens]* | Cell cycle, cell division, DNA synthesis, mitosis |
| Ssc.9627.1.A1_at | | 0,72 | 0,93 | 1,22 | *OVOL2* | 6,92E-03 | 1,15E-03 | 1,04E-04 | Zinc finger protein 339 | Transcription, transcription regulation |
| Ssc.21666.2.A1_at | | 0,85 | 0,87 | 1,20 | *PLA2G12B* | 9,29E-04 | 7,26E-04 | 3,76E-05 | Group XIIB secretory phospholipase A2-like protein precursor | Calcium ion binding, phospholipase A2 activity |
| Ssc.6877.1.A1_at | | 0,69 | 0,83 | 1,20 | *EXPH5* | 8,16E-04 | 1,60E-04 | 3,50E-06 | Slp homolog lacking C2 domains-b | Intracellular protein transport |
| Ssc.14503.1.S1_at | | 0,93 | 0,62 | 1,20 | *APOA4* | 1,38E-05 | 6,84E-04 | 8,30E-07 | Apolipoprotein A-IV precursor | Lipid transport, transport |
| Ssc.19270.1.S1_at | | 0,81 | 0,73 | 1,19 | *PTPRD* | 1,13E-02 | 2,00E-02 | 7,42E-04 | Protein-tyrosine phosphatase delta precursor | Hydrolase, protein phosphatase |
| Ssc.24018.1.S1_at | | 0,90 | 0,64 | 1,19 | *AKAP6* | 3,50E-04 | 5,00E-03 | 2,22E-05 | A-kinase anchor protein 6 | Protein targeting, receptor binding |
| Ssc.995.1.A1_at | | 0,44 | 0,74 | 1,19 | *CD163L1* | 6,68E-02 | 4,90E-03 | 1,03E-04 | Scavenger receptor cysteine-rich type 1 protein M160 precursor  *[H. sapiens]* | Scavenger receptor activity |
| Ssc.19564.1.S1_at | | 0,63 | 0,70 | 1,18 | *PCDH9* | 3,32E-02 | 2,05E-02 | 5,97E-04 | Protocadherin 9 precursor | Cell adhesion, calcium ion binding |
| Ssc.30330.1.A1_at | | 0,82 | 1,00 | 1,18 | *UHRF1BP1L* | 5,92E-03 | 1,49E-03 | 3,89E-04 | UHRF1-binding protein 1-like | - |
| Ssc.3112.1.A1_at | | 0,93 | 0,78 | 1,18 | *COBL* | 8,32E-04 | 3,27E-03 | 1,08E-04 | Cordon-bleu homolog *[H. sapiens]* | - |
| Ssc.13060.3.A1_a_at | | 0,77 | 0,68 | 1,17 | *PAPD5* | 7,45E-05 | 2,49E-04 | 8,07E-07 | PAP associated domain containing 5 *[H. sapiens]* | Cell cycle, cell division, DNA synthesis, mitosis |
| Ssc.18231.1.S1_at | | 0,21 | 0,38 | 1,17 | *RNF144A* | 3,31E-01 | 9,49E-02 | 6,95E-05 | Ubiquitin conjugating enzyme 7 interacting protein 4 | Ubl conjugation pathway |
| Ssc.13429.1.S1_at | | 0,19 | 0,51 | 1,17 | *YPEL2* | 3,83E-01 | 2,97E-02 | 6,88E-05 | - | - |

| Ssc.12990.1.A1_at | 0,50 | 0,48 | 1,17 | *MNAT1* | 5,17E-02 | 6,01E-02 | 2,15E-04 | CDK-activating kinase assembly factor MAT1 | Cell cycle, transcription, transcription regulation |
| --- | --- | --- | --- | --- | --- | --- | --- | --- | --- |
| Ssc.17877.1.A1_at | 0,85 | 0,72 | 1,16 | *NR3C2* | 1,91E-03 | 6,08E-03 | 1,37E-04 | Mineralocorticoid receptor | Receptor, protein binding |
| Ssc.11423.2.A1_at | 0,87 | 0,91 | 1,16 | *ORMDL1* | 4,39E-03 | 3,23E-03 | 4,86E-04 | ORM1-like 1 *[H. sapiens]* | Ceramide metabolic process |
| Ssc.29197.1.A1_at | 0,61 | 0,73 | 1,16 | *RASGEF1C* | 1,41E-02 | 4,78E-03 | 1,03E-04 | RasGEF domain family, member 1C *[H. sapiens]* | Guanine-nucleotide releasing factor |
| Ssc.7289.1.A1_at | 0,55 | 0,71 | 1,16 | *CTDSPL* | 2,81E-02 | 7,36E-03 | 1,57E-04 | CTD small phosphatase-like protein | Hydrolase, protein phosphatase |
| Ssc.2682.1.S1_at | 0,26 | 0,59 | 1,16 | *TSC22D3* | 1,98E-01 | 9,04E-03 | 3,58E-05 | Glucocorticoid-induced leucine zipper protein | Regulation of transcription, DNA-dependent |
| Ssc.21858.1.A1_at | 0,85 | 0,95 | 1,16 | *HSDL1* | 3,70E-05 | 1,11E-05 | 1,27E-06 | Steroid dehydrogenase-like  *[H. sapiens]* | Oxidoreductase activity, protein binding |
| Ssc.20303.1.S1_at | 0,86 | 0,65 | 1,16 | *PMM1* | 7,62E-04 | 6,31E-03 | 5,31E-05 | Phosphomannomutase 1 | Isomerase |
| Ssc.8805.1.A1_at | 0,60 | 0,89 | 1,15 | *NFIB* | 4,63E-02 | 5,95E-03 | 9,09E-04 | - | Activator |
| Ssc.26350.1.A1_at | 0,36 | 0,68 | 1,15 | *NLRP12* | 1,33E-01 | 8,60E-03 | 1,48E-04 | NACHT-, LRR- and PYD-containing protein 12 | ATP binding, caspase activator activity, protein binding |
| Ssc.30963.1.A1_at | 0,91 | 0,74 | 1,15 | *SLC6A4* | 1,69E-03 | 7,02E-03 | 2,46E-04 | Sodium-dependent serotonin transporter | Neurotransmitter transport, symport, transport, response to toxin |
| Ssc.6004.1.A1_at | 0,41 | 0,50 | 1,15 | *ZNF395* | 1,49E-02 | 4,53E-03 | 1,96E-06 | F-box only protein 16 | Transcription, transcription regulation |
| Ssc.21666.1.S1_at | 0,86 | 0,81 | 1,15 | *PLA2G12B* | 5,65E-04 | 8,56E-04 | 3,64E-05 | Group XIIB secretory phospholipase A2-like protein precursor | Calcium ion binding, phospholipase A2 activity |
| Ssc.2166.1.A1_at | 0,76 | 0,85 | 1,14 | *GCLC* | 1,27E-03 | 5,32E-04 | 3,18E-05 | Glutamate--cysteine ligase catalytic subunit | Ligaes, anti-apoptosis |
| Ssc.20352.2.S1_at | 0,66 | 0,95 | 1,14 | *RAB3GAP2* | 3,47E-03 | 1,79E-04 | 2,96E-05 | rab3 GTPase-activating protein, non-catalytic subunit *[H. sapiens]* | Intracellular protein transport, regulation of GTPase activity |
| Ssc.1046.1.A1_at | 0,78 | 0,91 | 1,14 | *UHRF1BP1L* | 7,19E-03 | 2,71E-03 | 4,41E-04 | - | - |
| Ssc.13145.1.A1_a_at | 0,61 | 0,56 | 1,14 | *CTDSPL* | 8,45E-04 | 1,62E-03 | 1,61E-06 | CTD small phosphatase-like protein | Hydrolase, protein phosphatase |
| Ssc.26624.1.S1_at | 0,94 | 0,84 | 1,14 | *PTER* | 1,97E-04 | 5,11E-04 | 2,92E-05 | Phosphotriesterase related protein | Hydrolase |
| Ssc.19338.1.A1_at | 0,70 | 0,78 | 1,14 | *DYNC2LI1* | 2,15E-02 | 1,22E-02 | 8,70E-04 | Dynein 2 light intermediate chain isoform 1 *[H. sapiens]* | Developmental protein, motor protein |
| Ssc.11404.1.A1_at | 0,66 | 0,70 | 1,14 | *ATXN1* | 1,86E-02 | 1,35E-02 | 4,27E-04 | Ataxin-1 | Protein binding, transcription repressor activity |
| Ssc.24226.1.A1_at | 0,81 | 0,61 | 1,12 | *PRKCA* | 1,41E-03 | 1,04E-02 | 8,15E-05 | Protein kinase C, alpha type | ATPase activity, receptor binding |
| Ssc.8003.1.S1_at | 0,69 | 0,80 | 1,12 | *MTMR4* | 7,48E-04 | 1,99E-04 | 6,25E-06 | Myotubularin related protein 4  *[H. sapiens]* | Hydrolase, protein phosphatase |
| Ssc.2396.1.A1_at | 0,78 | 0,87 | 1,12 | *ARL6IP2* | 2,32E-03 | 9,71E-04 | 1,05E-04 | ADP-ribosylation factor-like 6 interacting protein 2 *[H. sapiens]* | Hydrolase, GTPase activity, identical protein binding |
| Ssc.6106.2.S1_at | 0,85 | 0,59 | 1,12 | *PTPRD* | 1,70E-03 | 1,81E-02 | 1,71E-04 | Protein-tyrosine phosphatase delta precursor | Hydrolase, protein phosphatase, receptor, protein binding |
| Ssc.1554.1.S1_at | 0,31 | 0,50 | 1,12 | *ZNF395* | 1,28E-01 | 2,22E-02 | 4,74E-05 | F-box only protein 16 | Transcription, transcription regulation |
| Ssc.8833.1.S1_at | 0,91 | 0,95 | 1,12 | *IL15* | 1,22E-05 | 7,98E-06 | 1,23E-06 | Interleukin-15 precursor | Cytokine, immune response |
| Ssc.1384.2.S1_at | 0,73 | 0,77 | 1,12 | *AKAP9* | 2,16E-03 | 1,45E-03 | 5,44E-05 | A-kinase anchor protein 9 | Receptor binding, transport |
| Ssc.6995.1.A1_at | 0,62 | 0,99 | 1,12 | *ZBTB20* | 1,50E-02 | 5,79E-04 | 2,06E-04 | Zinc finger and BTB domain containing protein 20 | Transcription, transcription regulation |
| Ssc.5600.3.S1_a_at | 0,56 | 0,48 | 1,11 | *PPFIBP2* | 8,12E-04 | 2,73E-03 | 8,39E-07 | Liprin-beta 2 (Protein tyrosine phosphatase receptor type f polypeptide-interacting protein binding protein 2) | Protein binding |
| Ssc.8233.1.A1_at | 0,67 | 0,73 | 1,11 | *DNAJC6* | 5,32E-03 | 3,01E-03 | 8,99E-05 | DnaJ (Hsp40) homolog, subfamily C, member 6 | Chaperone, hydrolase, protein phosphatase |
| Ssc.206.1.S1_at | 0,93 | 0,70 | 1,11 | *CYP2C18* | 2,64E-03 | 1,56E-02 | 6,84E-04 | Cytochrome P450 2C18 | Monooxygenase, oxidoreductase |
| Ssc.3528.1.S1_at | 0,75 | 0,73 | 1,10 | *GOT1* | 1,26E-02 | 1,47E-02 | 9,53E-04 | Aspartate aminotransferase, cytoplasmic | Aminotransferase, transferase |
| Ssc.4729.1.S1_at | 0,59 | 0,48 | 1,10 | *LEAP2* | 1,95E-02 | 5,02E-02 | 2,51E-04 | Liver-expressed antimicrobial peptide 2 precursor | Transferase |
| Ssc.3394.3.A1_at | 0,66 | 0,57 | 1,10 | *CDR2* | 6,33E-03 | 1,54E-02 | 1,12E-04 | Cerebellar degeneration-related protein 2 | Protein binding |
| Ssc.29035.2.A1_at | 0,85 | 0,84 | 1,10 | *GCLC* | 3,98E-04 | 4,21E-04 | 3,54E-05 | Glutamate--cysteine ligase catalytic subunit | Ligase, anti-apoptosis |
| Ssc.30870.1.A1_at | 0,47 | 0,56 | 1,10 | *ANKRD37* | 7,78E-03 | 2,37E-03 | 4,66E-06 | Low density lipoprotein receptor-related protein binding protein *[H. sapiens]* | - |
| Ssc.18524.1.S1_at | 0,80 | 0,82 | 1,09 | *PNPLA2* | 1,02E-03 | 8,73E-04 | 6,51E-05 | Patatin-like phospholipase domain containing 2 *[H. sapiens]* | Hydrolase |
| Ssc.18245.1.A1_at | 0,39 | 0,47 | 1,09 | *BDH1* | 9,94E-02 | 4,96E-02 | 2,07E-04 | D-beta-hydroxybutyrate dehydrogenase, mitochondrial precursor | Oxidoreductase |
| Ssc.10232.1.A1_at | 0,47 | 0,74 | 1,09 | *MYLIP* | 2,84E-02 | 1,75E-03 | 5,63E-05 | Myosin regulatory light chain interacting protein *[H. sapiens]* | Ubl conjugation pathway |
| Ssc.6173.3.S1_a_at | 0,41 | 0,34 | 1,08 | *PDGFA* | 2,24E-02 | 5,41E-02 | 1,00E-05 | Platelet-derived growth factor, A chain precursor | Growth factor, mitogen |
| Ssc.24804.1.S1_at | 0,74 | 0,78 | 1,08 | *ZNF514* | 1,23E-03 | 7,59E-04 | 3,94E-05 | Zinc finger protein 514 | Transcription, transcription regulation |
| Ssc.30768.1.S1_at | 0,20 | 0,42 | 1,08 | *HMCN1* | 3,30E-01 | 5,19E-02 | 9,33E-05 | Hemicentin; fibulin 6 *[H. sapiens]* | Response to stimulus, calcium ion binding |
| Ssc.3394.1.A1_at | 0,64 | 0,42 | 1,07 | *CDR2* | 1,21E-02 | 7,98E-02 | 2,72E-04 | Cerebellar degeneration-related protein 2 | Protein binding |
| Ssc.11043.1.A1_at | 0,47 | 0,59 | 1,07 | *TEX2* | 2,69E-02 | 8,12E-03 | 6,90E-05 | Uncharacterized hypothalamus protein HT008 *[H. sapiens]* | Signal transduction |
| Ssc.25103.1.S1_at | 0,72 | 0,70 | 1,07 | *ABCG2* | 3,21E-03 | 3,79E-03 | 1,19E-04 | ATP-binding cassette, sub-family G, member 2 | Transport |
| Ssc.21942.3.A1_a_at | 0,48 | 0,51 | 1,06 | *TSKU* | 1,96E-02 | 1,30E-02 | 4,06E-05 | Tsukushi | Protein binding |
| Ssc.3068.1.S1_at | 0,82 | 0,75 | 1,06 | *IL11RA* | 5,23E-04 | 1,13E-03 | 4,88E-05 | Interleukin 11 receptor, alpha isoform 1 precursor *[H. sapiens]* | Cytokine receptor activity |
| Ssc.7598.1.A1_at | 0,47 | 0,58 | 1,06 | *PPAP2A* | 7,79E-03 | 1,70E-03 | 6,31E-06 | Lipid phosphate phosphohydrolase 1 | Hydrolase |
| Ssc.17368.1.S1_at | 0,47 | 0,42 | 1,05 | *ATG2B* | 1,19E-02 | 2,13E-02 | 1,60E-05 | - | - |
| Ssc.5600.1.S1_at | 0,53 | 0,59 | 1,05 | *PPFIBP2* | 3,36E-03 | 1,47E-03 | 6,15E-06 | Liprin-beta 2 | Protein binding |
| Ssc.3321.1.A1_at | 0,70 | 0,72 | 1,04 | *ALDH18A1* | 4,44E-03 | 3,75E-03 | 1,89E-04 | Delta 1-pyrroline-5-carboxylate synthetase | Kinase; oxidoreductase, transferase |
| Ssc.7004.1.A1_at | 0,69 | 0,79 | 1,04 | *ORMDL1* | 7,79E-03 | 3,26E-03 | 3,66E-04 | ORM1-like 1 *[H. sapiens]* | Ceramide metabolic process |
| Ssc.18924.1.A1_at | 0,48 | 0,88 | 1,03 | *DDX60* | 3,79E-02 | 8,77E-04 | 2,15E-04 | - | Helicase, hydrolase |
| Ssc.1414.1.A1_a_at | 0,67 | 0,58 | 1,03 | *NCOA1* | 8,15E-04 | 2,40E-03 | 1,34E-05 | Nuclear receptor coactivator 1 isoform 1 *[H. sapiens]* | Transcription, transcription regulation |
| Ssc.15582.1.S1_at | 0,63 | 0,65 | 1,03 | *ARHGAP18* | 1,86E-02 | 1,64E-02 | 7,17E-04 | Rho GTPase activating protein 18  *[H. sapiens]* | GTPase activation, protein binding |
| Ssc.11043.2.A1_at | 0,35 | 0,57 | 1,02 | *TEX2* | 5,85E-02 | 5,01E-03 | 3,25E-05 | Uncharacterized hypothalamus protein HT008 *[H. sapiens]* | Signal transduction, sphingolipid metabolic process |
| Ssc.4891.1.A1_at | 0,67 | 0,61 | 1,02 | *AGPAT9* | 2,09E-03 | 3,97E-03 | 5,32E-05 | Glycerol-3-phosphate acyltransferase 3 | Acetyltranferase, transferase, phospholipid biosynthesis |
| Ssc.4511.1.S1_at | 0,53 | 0,55 | 1,02 | *DHRS3* | 1,08E-02 | 8,96E-03 | 6,16E-05 | Short-chain dehydrogenase/reductase 3 | Oxidoreductase |
| Ssc.8663.1.A1_at | 0,65 | 0,74 | 1,02 | *PGRMC2* | 2,67E-03 | 9,30E-04 | 5,36E-05 | Membrane associated progesterone receptor component 2 | Receptor, steroid hormone receptor activity |
| Ssc.4707.1.A1_at | 0,34 | 0,68 | 1,02 | *KITLG* | 5,91E-02 | 1,26E-03 | 3,04E-05 | - | Growth factor |
| Ssc.8444.1.S1_at | 0,76 | 0,87 | 1,02 | *KIAA1109* | 2,32E-03 | 8,66E-04 | 2,22E-04 | PREDICTED: hypothetical protein KIAA1109 | Integral to membrane |
| Ssc.5940.1.S1_at | 0,85 | 0,73 | 1,02 | *C1orf115* | 2,85E-04 | 9,59E-04 | 5,06E-05 | - | - |
| Ssc.1796.1.A1_at | 0,59 | 0,63 | 1,02 | *DDHD2* | 3,56E-03 | 2,23E-03 | 3,37E-05 | DDHD domain containing 2 | Hydrolase |
| Ssc.21169.1.S1_at | 0,42 | 0,66 | 1,01 | *KLHL24* | 7,80E-02 | 1,02E-02 | 4,66E-04 | Kelch-like protein 24 | Protein binding |
| Ssc.6379.1.A1_at | 0,65 | 0,59 | 1,01 | *OCLN* | 2,38E-04 | 5,67E-04 | 2,29E-06 | Occludin | Protein binding |
| Ssc.5491.1.S1_at | 0,56 | 0,59 | 1,01 | *PTGES3* | 2,52E-03 | 1,70E-03 | 1,17E-05 | Telomerase-binding protein p23 | Fatty acid biosynthesis, lipid synthesis, prostaglandin biosynthesis |
| Ssc.10047.1.A1_at | 0,72 | 0,63 | 1,01 | *BRI3P1* | 2,77E-04 | 8,39E-04 | 9,32E-06 | Brain protein I3 | Integral to membrane |
| Ssc.5407.1.A1_at | 0,60 | 0,66 | 1,01 | *FAM134B* | 1,37E-03 | 6,33E-04 | 1,03E-05 | Protein FAM134B | sensory perception of pain |
| Ssc.25021.1.S1_at | 0,55 | 0,66 | 1,01 | *UBA5* | 4,88E-03 | 1,43E-03 | 2,85E-05 | Ubiquitin-activating enzyme E1-domain containing 1 isoform 1  *[H. sapiens]* | Ubl conjugation pathway |
| Ssc.9546.1.S1_at | 0,31 | 0,48 | 1,01 | *ADO* | 7,48E-02 | 1,07E-02 | 2,37E-05 | - | - |
| Ssc.14376.1.A1_at | 0,66 | 0,72 | 1,01 | *SSX2IP* | 2,64E-03 | 1,41E-03 | 7,75E-05 | Synovial sarcoma, X breakpoint 2 interacting protein  *[H. sapiens]* | Cell adhesion |
| Ssc.28780.1.A1_at | 0,42 | 0,59 | 1,00 | *KLHL7* | 8,05E-02 | 1,82E-02 | 4,57E-04 | SBBI26 protein *[H. sapiens]* | Protein binding |
| Ssc.25221.1.A1_at | 0,86 | 0,86 | 1,00 | *CDC14B* | 1,01E-03 | 9,88E-04 | 2,68E-04 | CDC14 homolog B isoform 1  *[H. sapiens]* | Protein binding |
| Ssc.18385.1.S1_at | 0,33 | 0,31 | 1,00 | *ABAT* | 1,46E-01 | 1,78E-01 | 3,72E-04 | 4-aminobutyrate aminotransferase, mitochondrial precursor | Neurotransmitter degradation |
| Ssc.22342.1.A1_s_at | -0,70 | -0,60 | -1,00 | *SRD5A2* | 4,46E-03 | 1,17E-02 | 2,62E-04 | 3-oxo-5-alpha-steroid 4-dehydrogenase 2 | Oxidoreductase, differentiation |
| Ssc.6376.1.A1_at | -0,29 | -0,25 | -1,01 | *C10orf68* | 9,40E-02 | 1,41E-01 | 2,11E-05 | Coiled-coil domain containing protein 7 | - |
| Ssc.18456.2.S1_at | -0,33 | -0,61 | -1,01 | *ETS2* | 7,70E-02 | 3,45E-03 | 4,80E-05 | C-ets-2 protein | Protein binding |
| Ssc.11395.1.S1_at | -0,49 | -0,58 | -1,01 | *PI4K2A* | 2,75E-02 | 1,11E-02 | 1,84E-04 | Phosphatidylinositol 4-kinase type II *[H. sapiens]* | Kinase, transferase |
| Ssc.6972.1.A1_at | -0,64 | -0,60 | -1,01 | *HSPA13* | 1,89E-02 | 2,67E-02 | 9,95E-04 | Microsomal stress 70 protein ATPase core precursor | ATP binding |
| Ssc.6514.3.S1_a_at | -0,28 | -0,56 | -1,01 | *GALE* | 2,60E-01 | 3,80E-02 | 9,69E-04 | UDP-glucose 4-epimerase | Isomerase |
| Ssc.30800.1.A1_at | -0,45 | -0,72 | -1,02 | *-* | 3,34E-02 | 2,12E-03 | 1,09E-04 | - | - |
| Ssc.8974.1.S1_at | -0,72 | -0,76 | -1,02 | *CPT1A* | 1,84E-03 | 1,34E-03 | 9,27E-05 | Carnitine O-palmitoyltransferase I, mitochondrial liver isoform | Acyltransferase, transferase |
| Ssc.20.1.S1_at | -0,46 | -0,63 | -1,03 | *IL18* | 6,04E-02 | 1,41E-02 | 4,47E-04 | Interleukin-18 precursor | Cytokine, signal transducer activity |
| Ssc.15492.1.S1_at | -0,44 | -0,90 | -1,03 | *TRIB1* | 4,65E-02 | 5,02E-04 | 1,60E-04 | G-protein-coupled receptor induced protein *[H. sapiens]* | Protein kinase inhibitor |
| Ssc.22097.1.A1_at | -0,46 | -0,64 | -1,03 | *EYA3* | 5,14E-02 | 9,77E-03 | 3,00E-04 | Eyes absent homolog 3 | Activator, chromatin regulator, developmental protein, hydrolase, protein phosphatase |
| Ssc.12452.1.S1_at | -0,81 | -0,48 | -1,03 | *MYO1B* | 4,45E-03 | 6,22E-02 | 6,98E-04 | Myosin Ib | Motor protein, myosine |
| Ssc.13646.1.A1_at | 0,08 | -0,20 | -1,03 | *ENTPD7* | 7,32E-01 | 3,77E-01 | 4,16E-04 | Ectonucleoside triphosphate diphosphohydrolase 7 *[H. sapiens]* | Hydrolase |
| Ssc.8827.1.A1_at | -0,96 | -0,74 | -1,04 | *CREB1* | 9,63E-05 | 9,92E-04 | 4,29E-05 | cAMP response element binding protein | Activator |
| Ssc.31164.1.S1_at | -0,82 | -0,44 | -1,04 | *MYO1B* | 4,59E-03 | 9,01E-02 | 7,48E-04 | Myosin Ib | Motor protein, myosine |
| Ssc.26925.1.S1_at | -0,50 | -0,66 | -1,04 | *ZNF677* | 3,51E-04 | 2,65E-05 | 1,37E-07 | Zinc finger protein 677 | Transcription, trnascription regulation |
| Ssc.28645.1.A1_at | -0,75 | -0,66 | -1,04 | *SLC7A1* | 6,21E-04 | 1,75E-03 | 2,67E-05 | High affinity cationic amino acid transporter 1 | Receptor |
| Ssc.2634.1.S1_at | -0,40 | -0,72 | -1,04 | *C14orf21* | 8,97E-02 | 6,07E-03 | 3,39E-04 | Leukotriene B4 receptor 2 (LTB4-R2) | RNA binding |
| Ssc.22038.1.S1_at | -0,58 | -0,82 | -1,05 | *DNAJB11* | 2,77E-02 | 3,44E-03 | 5,43E-04 | DnaJ homolog subfamily B member 11 precursor | Chaperone, protein folding, heat shock protein binding |
| Ssc.14326.1.A1_at | -0,53 | -0,54 | -1,05 | *MAPK13* | 5,16E-02 | 4,52E-02 | 8,51E-04 | Mitogen-activated protein kinase 13 | Cell cycle, response to stress, protein binding |
| Ssc.15638.1.A1_at | -0,29 | -0,45 | -1,05 | *AMPD3* | 1,51E-01 | 3,21E-02 | 7,66E-05 | AMP deaminase 3 | Hydrolase |
| Ssc.23797.1.S1_at | -0,58 | -0,68 | -1,05 | *CCL4L* | 2,36E-02 | 1,03E-02 | 4,41E-04 | Chemokine (C-C motif) ligand 4-like precursor | Chemokine, chemotaxis, immune response, inflammatory response |
| Ssc.2847.1.S1_at | -0,24 | -0,39 | -1,05 | *ANKRD9* | 2,36E-01 | 6,21E-02 | 8,92E-05 | Ankyrin repeat domain 9 | - |
| Ssc.28517.1.S1_at | -0,82 | -0,78 | -1,06 | *DUSP10* | 4,42E-04 | 6,72E-04 | 4,25E-05 | Dual specificity protein phosphatase 10 | Hydrolase, protein phosphatase |
| Ssc.21253.1.S1_at | -0,28 | -0,54 | -1,06 | *SPDEF* | 1,87E-01 | 1,67E-02 | 1,13E-04 | SAM pointed domain containing ets transcription factor [*H. sapiens]* | Activator, transcription, transcription regulation |
| Ssc.29841.1.A1_s_at | -0,74 | -0,66 | -1,06 | *UGT2B11* | 4,42E-03 | 9,10E-03 | 2,68E-04 | UDP-glucuronosyltransferase 2B11 precursor, microsomal | Glycosyltransferase, transferase |
| Ssc.15765.2.S1_a_at | -0,79 | -0,80 | -1,07 | *CD69* | 1,34E-03 | 1,18E-03 | 9,92E-05 | Early activation antigen CD69 | Sugar binding, transmembrane receptor activity |
| Ssc.29841.1.A1_at | -0,89 | -0,82 | -1,07 | *UGT2B11* | 2,15E-03 | 3,76E-03 | 4,93E-04 | UDP-glucuronosyltransferase 2B11 precursor, microsomal | Glycosyltransferase, transferase |
| Ssc.314.1.S1_at | -0,20 | -0,49 | -1,07 | *ADM* | 1,22E-01 | 1,08E-03 | 4,08E-07 | ADM precursor | Hormone, response to wounding |
| Ssc.6193.1.A1_at | -0,53 | -0,61 | -1,07 | *RAB24* | 3,13E-02 | 1,50E-02 | 2,72E-04 | Ras-related protein Rab-24 | Autophagy, protein transport, transport |
| Ssc.6586.2.A1_a_at | -0,48 | -0,71 | -1,07 | *CHIC2* | 4,52E-04 | 9,99E-06 | 7,81E-08 | Cysteine-rich hydrophobic domain 2 *[H. sapiens]* | Protein binding |
| Ssc.27215.1.S1_a_at | -0,43 | -0,52 | -1,08 | *ALS2CL* | 2,25E-02 | 8,03E-03 | 1,77E-05 | ALS2 C-terminal like isoform 1  *[H. sapiens]* | GTPase activation, identical protein binding |
| Ssc.6822.1.A1_at | -0,36 | -0,38 | -1,08 | *NCOA7* | 1,01E-01 | 7,99E-02 | 1,09E-04 | Nuclear receptor coactivator 7 *[H. sapiens]* | Transcription, transcription regulation, protein binding, activator |
| Ssc.9790.1.S1_at | -0,45 | -0,72 | -1,08 | *DDX3X* | 2,20E-03 | 3,05E-05 | 3,48E-07 | DEAD-box protein 3 | Host-virus interaction |
| Ssc.1303.1.S1_at | -0,60 | -0,81 | -1,08 | *SERPINB1* | 2,70E-02 | 5,30E-03 | 5,86E-04 | Leukocyte elastase inhibitor | Protease inhibitor, serine protease inhibitor |
| Ssc.30613.1.A1_at | -0,44 | -0,47 | -1,09 | *ARL4A* | 1,08E-01 | 8,64E-02 | 8,31E-04 | ADP-ribosylation factor-like protein 4A | GTP binding, protein binding |
| Ssc.20071.1.S1_at | -0,74 | -0,99 | -1,09 | *ARMET* | 1,12E-02 | 1,55E-03 | 7,50E-04 | ARMET protein precursor | Growth factor, unfolded protein response |
| Ssc.21959.1.S1_at | -0,61 | -0,68 | -1,10 | *CARS* | 9,27E-03 | 4,68E-03 | 9,58E-05 | Cysteinyl-tRNA synthetase | Protein biosynthesis |
| Ssc.2087.1.S1_at | -0,65 | -0,94 | -1,10 | *HYOU1* | 1,58E-02 | 1,45E-03 | 3,83E-04 | 150 kDa oxygen-regulated protein precursor | Stress response, protein binding, chaperone |
| Ssc.1696.1.A1_at | -0,40 | -0,38 | -1,11 | *FMNL2* | 8,31E-02 | 9,32E-02 | 1,31E-04 | Formin-like 2 isoform D *[H. sapiens]* | Actin binding |
| Ssc.21290.1.S1_at | -0,39 | -0,73 | -1,11 | *TSC22D2* | 5,00E-02 | 1,34E-03 | 2,96E-05 | - | Regulation of transcription, DNA-dependent |
| Ssc.1509.1.S1_at | -0,38 | -0,70 | -1,11 | *TNFRSF21* | 1,11E-02 | 1,09E-04 | 7,31E-07 | Tumor necrosis factor receptor superfamily member 21 precursor | Receptor, protein binding, apoptosis |
| Ssc.6163.2.S1_at | -0,36 | -0,74 | -1,11 | *ETS2* | 8,81E-02 | 2,24E-03 | 6,96E-05 | C-ets-2 protein | Positive regulation of transcription, DNA-dependent, protein binding |
| Ssc.25376.1.S1_at | -0,43 | -0,26 | -1,11 | *C14orf38* | 9,13E-02 | 2,89E-01 | 3,81E-04 | 50 kDa protein | - |
| Ssc.1143.3.S1_a_at | -0,27 | -0,48 | -1,12 | *ANXA4* | 2,16E-01 | 3,82E-02 | 1,08E-04 | Annexin A4 | Anti-apoptosis, calcium ion binding |
| Ssc.13462.1.A1_at | 0,08 | -0,19 | -1,12 | *TBC1D9* | 7,69E-01 | 4,66E-01 | 6,39E-04 | - | GTPase activation, calcium ion binding |
| Ssc.2062.1.A1_at | -0,32 | -0,57 | -1,13 | *BZW2* | 1,20E-01 | 1,14E-02 | 4,89E-05 | Basic leucine zipper and W2 domains 2 *[H. sapiens]* | Developmental protein, protein binding |
| Ssc.4807.1.S1_at | -0,61 | -0,77 | -1,13 | *DEAF1* | 2,90E-02 | 8,40E-03 | 4,63E-04 | Deformed epidermal autoregulatory factor 1 homolog | Developmental protein, protein binding |
| Ssc.25098.1.S1_at | -0,49 | -0,52 | -1,13 | *MPP6* | 1,91E-02 | 1,28E-02 | 2,50E-05 | MAGUK p55 subfamily member 6 | Protein binding |
| Ssc.27027.1.S1_at | -0,43 | -0,46 | -1,13 | *ZC3H12A* | 7,09E-02 | 5,63E-02 | 1,51E-04 | - | Angiogenesis, apoptosis, differentiation |
| Ssc.3088.1.S1_at | -0,60 | -0,62 | -1,14 | *MRC2* | 1,31E-02 | 1,17E-02 | 1,04E-04 | Mannose receptor, C type 2; endocytic receptor (macrophage mannose receptor family)  *[H. sapiens]* | Receptor |
| Ssc.9951.1.A1_at | -0,60 | -0,71 | -1,15 | *GNPNAT1* | 1,32E-02 | 4,79E-03 | 8,80E-05 | Glucosamine 6-phosphate N-acetyltransferase | Acyltransferase, transferase |
| Ssc.11571.3.S1_at | -0,11 | -0,36 | -1,15 | *FAM118B* | 6,60E-01 | 1,52E-01 | 2,55E-04 | Protein FAM118B | - |
| Ssc.27493.1.S1_at | -0,62 | -0,55 | -1,15 | *NDUFS1* | 1,93E-03 | 4,62E-03 | 5,71E-06 | NADH-ubiquinone oxidoreductase 75 kDa subunit, mitochondrial precursor | Transport, apoptosis, protein binding |
| Ssc.23937.1.A1_at | -0,34 | -0,64 | -1,16 | *SLCO1B1* | 4,73E-02 | 1,17E-03 | 3,59E-06 | Solute carrier organic anion transporter family member 1B1 | Ion transport, transport |
| Ssc.27512.1.A1_at | -0,59 | -0,85 | -1,16 | *RNF123* | 1,69E-02 | 1,60E-03 | 1,07E-04 | Ring finger protein 123 *[H. sapiens]* | Ubl conjugation pathway |
| Ssc.10208.1.A1_s_at | -0,70 | -0,75 | -1,17 | *OBFC2A* | 2,02E-04 | 1,08E-04 | 9,69E-07 | - | DNA damage, DNA repair, single-stranded DNA binding |
| Ssc.29842.1.A1_at | -0,33 | -0,34 | -1,17 | *GPD2* | 1,15E-01 | 1,05E-01 | 4,02E-05 | Glycerol-3-phosphate dehydrogenase, mitochondrial precursor | Oxidoreductase, calcium ion binding |
| Ssc.27401.1.S1_at | -0,53 | -0,59 | -1,18 | *CELSR1* | 3,93E-02 | 2,45E-02 | 1,74E-04 | Cadherin EGF LAG seven-pass G-type receptor 1 precursor | Developmental protein, G-protein coupled receptor, receptor, transducer |
| Ssc.1271.2.A1_at | -0,71 | -0,75 | -1,18 | *FAM152A* | 1,38E-03 | 9,74E-04 | 1,29E-05 | Protein CGI-96 | - |
| Ssc.5204.1.S1_at | -0,42 | -0,61 | -1,18 | *CDA* | 9,14E-02 | 1,95E-02 | 1,76E-04 | Cytidine deaminase | Hydrolase |
| Ssc.1555.1.A1_at | -0,45 | -0,63 | -1,18 | *FOS* | 1,40E-02 | 1,58E-03 | 3,82E-06 | Proto-oncogene protein c-fos | Cellular response to reactive oxygen species, inflammatory response |
| Ssc.12927.1.S1_at | -0,21 | -0,46 | -1,18 | *HIGD1A* | 2,26E-01 | 1,52E-02 | 5,98E-06 | - | Stress response, protein binding |
| Ssc.5650.1.S1_at | -0,43 | -0,67 | -1,18 | *ELOVL1* | 5,26E-02 | 5,64E-03 | 4,87E-05 | Elongation of very long chain fatty acids protein 1 | Fatty acid biosynthesis, lipi synthesis, protein binding |
| Ssc.17423.1.S1_at | -0,52 | -0,59 | -1,19 | *YWHAZ* | 3,97E-03 | 1,49E-03 | 1,59E-06 | Tyrosine 3/tryptophan 5 -monooxygenase activation protein, zeta polypeptide *[H. sapiens]* | Transcription factor binding |
| Ssc.4999.1.S1_at | -0,48 | -0,83 | -1,19 | *AVPI1* | 8,25E-02 | 5,86E-03 | 3,95E-04 | Vasopressin-induced protein, 32kDa *[H. sapiens]* | Cell cycle |
| Ssc.3719.1.S1_at | -0,55 | -0,64 | -1,20 | *TIFA* | 4,25E-02 | 2,04E-02 | 2,49E-04 | TRAF2 binding protein *[H. sapiens]* | Protein binding |
| Ssc.29067.1.A1_s_at | -0,43 | -0,63 | -1,22 | *BZW2* | 6,12E-02 | 9,59E-03 | 4,33E-05 | Basic leucine zipper and W2 domains 2 *[H. sapiens]* | Cell differentiation, protein binding |
| Ssc.17821.1.A1_at | -0,79 | -0,81 | -1,22 | *FAM135A* | 3,91E-03 | 3,53E-03 | 1,11E-04 | - | - |
| Ssc.22104.1.A1_at | -0,82 | -0,91 | -1,23 | *SLC23A2* | 8,02E-04 | 3,15E-04 | 1,70E-05 | Solute carrier family 23, member 2 | Ion transport, sodium transport, symport, transport |
| Ssc.6357.1.S1_at | -0,57 | -0,50 | -1,23 | *KCNK1* | 7,19E-02 | 1,09E-01 | 8,47E-04 | Potassium channel subfamily K member 1 | Ionic channel, potassium channel, voltage-gated channel |
| Ssc.9770.1.A1_at | -0,64 | -0,77 | -1,24 | *SEC23B* | 3,28E-03 | 7,40E-04 | 7,88E-06 | Protein transport protein Sec23B | ER-Golgi transport, protein transport, transport, protein binding |
| Ssc.8300.1.A1_at | -0,60 | -0,46 | -1,25 | *TIP1* | 1,06E-02 | 4,21E-02 | 2,91E-05 | TGF beta-inducible nuclear protein 1 | Ribonucleoprotein, rRNA processing |
| Ssc.30320.1.S1_at | -0,45 | -0,33 | -1,25 | *EPB41L4A* | 7,24E-02 | 1,77E-01 | 1,03E-04 | Band 4.1-like protein 4A | cytoskeletal protein binding |
| Ssc.27015.1.A1_at | -0,38 | -0,48 | -1,25 | *C10orf68* | 1,32E-01 | 5,88E-02 | 1,10E-04 | Coiled-coil domain containing protein 7 | - |
| Ssc.9016.1.A1_at | -0,25 | -0,77 | -1,26 | *SOCS1* | 2,49E-01 | 2,57E-03 | 3,31E-05 | Suppressor of cytokine signaling 1 | Ubl conjugation pathway, growth regulation |
| Ssc.888.1.A1_at | -0,39 | -0,90 | -1,27 | *ERRFI1* | 4,28E-02 | 1,32E-04 | 3,87E-06 | Mitogen-inducible gene 6 protein | Response to stress, Rho GTPase activator activity, protein kinase binding |
| Ssc.15576.1.S1_at | -0,60 | -0,92 | -1,27 | *CEBPG* | 2,69E-03 | 7,05E-05 | 2,18E-06 | CCAAT/enhancer binding protein gamma | Transcription, transcription regulator, activator |
| Ssc.10552.1.A1_at | -1,14 | -0,86 | -1,28 | *PTPRG* | 1,02E-05 | 1,74E-04 | 2,97E-06 | Protein-tyrosine phosphatase gamma precursor | Hydrolase, protein phosphatase |
| Ssc.376.1.S1_at | -0,28 | -0,52 | -1,28 | *HAMP* | 3,66E-01 | 1,10E-01 | 8,78E-04 | Hepcidin precursor (Liver-expressed antimicrobial peptide) | Defense response to bacterium, immune response, antibiotic, antimicrobial, fungicide, hormone |
| Ssc.11746.1.A1_at | -0,57 | -0,79 | -1,28 | *Q7Z4C3* | 5,85E-04 | 2,80E-05 | 1,14E-07 | MSTP150 | - |
| Ssc.9262.1.A1_at | -0,76 | -0,93 | -1,28 | *HRH1* | 2,31E-03 | 4,37E-04 | 2,00E-05 | Histamine H1 receptor | Inflammatory response, activation of phospholipase C activity by G-protein coupled receptor protein signaling pathway coupled to IP3 second messenger, histamine receptor activity |
| Ssc.4929.1.S1_at | -0,72 | -0,90 | -1,29 | *STK40* | 1,21E-03 | 1,76E-04 | 4,25E-06 | SINK-homologous serine/threonine kinase *[H. sapiens]* | Kinase, serine/threonine-protein kinase, transferase |
| Ssc.5737.1.S1_at | -0,61 | -0,96 | -1,30 | *CDKN1A* | 3,73E-03 | 8,58E-05 | 3,62E-06 | Cyclin-dependent kinase inhibitor 1 (p21) | Cell cycle, protein kinase inhibitor |
| Ssc.27799.1.S1_at | -0,47 | -0,77 | -1,33 | *EFNB2* | 1,09E-01 | 1,35E-02 | 2,52E-04 | Ephrin-B2 precursor (EPH-related receptor tyrosine kinase ligand 5) | Differentiation, host-virus interaction, neurogenesis |
| Ssc.15615.1.S1_a_at | -0,68 | -0,71 | -1,34 | *TARS* | 6,56E-03 | 4,99E-03 | 1,86E-05 | Threonyl-tRNA synthetase, cytoplasmic | Protein biosynthesis |
| Ssc.18470.1.A1_at | -1,03 | -0,94 | -1,35 | *PLXDC1* | 3,29E-03 | 6,19E-03 | 3,81E-04 | Plexin domain containing 1 precursor *[H. sapiens]* | Angiogenesis |
| Ssc.18757.1.S1_at | 0,25 | -0,81 | -1,39 | *C7orf53* | 4,30E-01 | 1,87E-02 | 4,33E-04 | Coiled-coil domain-containing transmembrane protein C7orf53 | Integral to membrane |
| Ssc.21926.1.S1_at | -0,44 | -0,89 | -1,39 | *LDLR* | 2,75E-02 | 2,13E-04 | 2,14E-06 | Low-density lipoprotein receptor precursor (LDL receptor) | Receptor, protein binding, calcium ion binding |
| Ssc.4425.1.S1_at | -0,56 | -0,37 | -1,39 | *PCDH15* | 1,01E-01 | 2,61E-01 | 6,41E-04 | Protocadherin 15 precursor | Response to stimulus, calcium ion binding |
| Ssc.22567.1.S1_at | -0,21 | -0,63 | -1,41 | *ENTPD7* | 2,99E-01 | 6,19E-03 | 5,13E-06 | Ectonucleoside triphosphate diphosphohydrolase 7 *[H. sapiens]* | Hydrolase |
| Ssc.25840.1.S1_at | -0,48 | -0,41 | -1,46 | *EHF* | 1,00E-01 | 1,56E-01 | 1,13E-04 | ets homologous factor; epithelium-specific ets factor 3 *[H. sapiens]* | Transcription, trannscription regulation, protein binding, epithelial cell differentiation |
| Ssc.22631.1.S1_at | -0,46 | -0,62 | -1,46 | *GFPT1* | 1,16E-01 | 4,21E-02 | 1,11E-04 | Glucosamine--fructose-6-phosphate aminotransferase [isomerizing] 1 | Aminotransferase, transferase |
| Ssc.22090.1.A1_at | -0,79 | -0,83 | -1,47 | *HS3ST1* | 2,60E-04 | 1,78E-04 | 3,74E-07 | Heparan sulfate D-glucosaminyl 3-O-sulfotransferase 1 precursor  *[H. sapiens]* | Transferase |
| Ssc.3712.1.S1_at | -0,60 | -0,48 | -1,49 | *ANKRD55* | 1,21E-02 | 3,40E-02 | 4,75E-06 | Ankyrin repeat domain-containing protein 55 | - |
| Ssc.196.1.S1_at | -1,55 | -1,21 | -1,49 | *PLAT* | 3,11E-04 | 2,36E-03 | 4,41E-04 | Tissue-type plasminogen activator precursor | Protein binding, hydrolase, protease, serine protease |
| Ssc.6159.1.A1_at | -0,63 | -0,90 | -1,49 | *TRAPPC9* | 3,59E-02 | 5,17E-03 | 7,86E-05 | T1 protein *[H. sapiens]* | Differentiation |
| Ssc.26201.1.S1_at | -1,10 | -1,11 | -1,50 | *SCGB2A1* | 4,53E-03 | 4,40E-03 | 4,24E-04 | Mammaglobin B precursor | Androgen binding |
| Ssc.26216.2.A1_at | -0,14 | -0,62 | -1,52 | *SOCS1* | 5,51E-01 | 1,82E-02 | 1,42E-05 | Suppressor of cytokine signaling 1 | Growth regulation, Ubl conjugation pathway |
| Ssc.10243.1.A1_at | -0,99 | -1,04 | -1,56 | *CSMD3* | 2,68E-03 | 1,79E-03 | 4,95E-05 | CUB and sushi multiple domains protein 3 precursor | - |
| Ssc.15927.1.A1_at | -1,14 | -1,02 | -1,56 | *MMP3* | 1,92E-03 | 4,35E-03 | 1,30E-04 | Stromelysin-1 precursor; Matrix metalloproteinase-3 | Proteolysis, metalloendopeptidase activity |
| Ssc.28567.1.A1_at | -0,63 | -0,67 | -1,57 | *PGM2* | 8,12E-03 | 5,48E-03 | 2,49E-06 | Phosphoglucomutase 2 *[H. sapiens]* | Isomerase |
| Ssc.13273.1.A1_at | -0,28 | -0,50 | -1,59 | *GCNT3* | 3,41E-01 | 9,79E-02 | 6,05E-05 | Glucosaminyl (N-acetyl) transferase 3, mucin type  *[H. sapiens]* | Glycosyltransferase, transferase |
| Ssc.1870.1.S1_at | -0,52 | -0,69 | -1,61 | *C1GALT1* | 5,63E-02 | 1,51E-02 | 1,44E-05 | Core 1 UDP-galactose:N-acetylgalactosamine-alpha-R beta 1,3-galactosyltransferase  *[H. sapiens]* | Developmental protein, glycosyltransferase, transferase |
| Ssc.9972.1.A1_at | -0,86 | -0,97 | -1,63 | *SEMA3G* | 8,34E-04 | 2,95E-04 | 1,29E-06 | Semaphorin sem2 *[H. sapiens]* | Receptor activity |
| Ssc.16844.1.A1_at | -0,27 | -0,18 | -1,66 | *TC2N* | 3,27E-01 | 5,19E-01 | 2,56E-05 | Membrane targeting tandem C2 domain containing protein 1 | - |
| Ssc.6323.1.S1_at | -0,87 | -0,81 | -1,67 | *ADFP* | 3,94E-05 | 7,40E-05 | 1,96E-08 | Adipophilin | - |
| Ssc.25242.1.A1_at | -0,72 | -0,58 | -1,68 | *EHF* | 6,99E-02 | 1,41E-01 | 4,62E-04 | ets homologous factor; epithelium-specific ets factor 3 *[H. sapiens]* | Transcription, trannscription regulation, protein binding, epithelial cell differentiation |
| Ssc.9796.1.A1_at | -0,82 | -1,28 | -1,71 | *PHLDA2* | 2,14E-02 | 1,23E-03 | 9,85E-05 | Pleckstrin homology-like domain family A member 2 | Apoptosis |
| Ssc.16218.1.S1_at | -0,67 | -0,84 | -1,75 | *ITGA2* | 9,62E-02 | 4,26E-02 | 3,77E-04 | Integrin alpha-2 precursor | Receptor, cell adhesion, host-virus interaction |
| Ssc.9938.1.A1_at | -0,50 | -0,58 | -1,75 | *GPR120* | 1,05E-01 | 6,50E-02 | 2,84E-05 | G protein-coupled receptor 120  *[H. sapiens]* | G-protein coupled receptor, receptor, transducer |
| Ssc.10168.1.A1_at | -0,50 | -1,11 | -1,76 | *ENC1* | 1,72E-01 | 6,36E-03 | 1,76E-04 | Ectoderm-neural cortex-1 protein | Ubl conjugation pathway |
| Ssc.14467.2.S1_a_at | -0,26 | -0,96 | -1,80 | *AREG* | 3,52E-01 | 3,50E-03 | 1,29E-05 | Amphiregulin precursor (AR) | Cytokine, growth factor, G-protein coupled receptor protein signaling pathway |
| Ssc.16819.1.A1_at | -0,70 | -1,05 | -1,81 | *ENC1* | 2,26E-02 | 1,90E-03 | 1,23E-05 | Ectoderm-neural cortex-1 protein | Ubl conjugation pathway |
| Ssc.21663.1.A1_at | -1,36 | -1,42 | -1,82 | *LIPG* | 1,88E-03 | 1,34E-03 | 1,55E-04 | Endothelial lipase precursor | Hydrolase, protein binding |
| Ssc.658.1.S1_at | -0,31 | -0,66 | -1,82 | *IL8* | 3,75E-01 | 6,68E-02 | 8,67E-05 | Interleukin-8 precursor | Cytokine, immune response, inflammatory response, chemotaxis |
| Ssc.4985.1.A1_at | -0,69 | -0,77 | -1,86 | *ITGA2* | 2,74E-02 | 1,57E-02 | 1,11E-05 | Integrin alpha-2 | Receptor, cell adhesion, host-virus interaction |
| Ssc.384.2.S1_at | -0,36 | -0,76 | -1,94 | *GFPT1* | 2,16E-01 | 1,78E-02 | 7,88E-06 | Glucosamine--fructose-6-phosphate aminotransferase [isomerizing] 1 | Aminotransferase, transferase |
| Ssc.16250.1.S2_at | -1,37 | -1,63 | -2,01 | *IL1RN* | 5,11E-03 | 1,49E-03 | 2,64E-04 | Interleukin-1 receptor antagonist protein precursor | Cytokine activity, interleukin-1 receptor antagonist activity, immune response, inflammatory response |
| Ssc.16013.1.S1_at | -1,00 | -0,94 | -2,05 | *MMP1* | 6,83E-03 | 1,03E-02 | 1,55E-05 | Interstitial collagenase precursor | Proteolysis, metalloendopeptidase activity |
| Ssc.30857.1.S1_at | -0,80 | -1,12 | -2,05 | *ENC1* | 2,60E-02 | 3,61E-03 | 1,75E-05 | Ectoderm-neural cortex-1 protein | Ubl conjugation pathway |
| Ssc.19907.1.S1_at | -1,55 | -1,55 | -2,22 | *F3* | 2,53E-03 | 2,57E-03 | 1,26E-04 | Tissue factor precursor | Blood coagulation |
| Ssc.11609.1.A1_at | -1,10 | -1,15 | -2,28 | *ASNS* | 1,60E-02 | 1,26E-02 | 5,75E-05 | Asparagine synthetase [glutamine-hydrolyzing] | Ligase |
| Ssc.33.1.S1_at | -1,22 | -0,99 | -2,33 | *DUOX2* | 1,63E-03 | 7,01E-03 | 3,34E-06 | Dual oxidase 2 precursor | Oxidoreductase, peroxidase, cytokine-mediated signaling pathway |
| Ssc.29281.1.A1_at | -1,47 | -1,35 | -2,35 | *SLC7A11* | 3,75E-03 | 6,41E-03 | 6,92E-05 | Cystine/glutamate transporter | Response to toxin, transport |
| Ssc.18918.1.A1_at | -0,92 | -1,01 | -2,43 | *GPX2* | 2,21E-02 | 1,35E-02 | 9,34E-06 | Glutathione peroxidase-gastrointestinal | Oxidoreductase, peroxidase, response to oxidative stress |
| Ssc.6189.1.A1_at | -1,64 | -1,52 | -2,90 | *SLC7A11* | 1,48E-03 | 2,60E-03 | 6,77E-06 | Cystine/glutamate transporter | Response to toxin, transport |
| Ssc.16470.1.S1_a_at | -0,12 | -0,48 | -3,10 | *REG3A (PAP)* | 7,19E-01 | 1,64E-01 | 1,82E-07 | Pancreatitis-associated protein 1 precursor | Acute phase, inflammatory response |
| Ssc.196.1.S1_at | -1,55 | -1,21 | -1,49 | *PLAT* | 3,11E-04 | 2,36E-03 | 4,41E-04 | Tissue-type plasminogen activator precursor |  |
| Ssc.9311.1.A1_at | -1,22 | -0,92 | -0,92 | *PHLDA1* | 7,85E-05 | 9,53E-04 | 1,01E-03 | Pleckstrin homology-like domain, family A, member 1 | Apoptosis, protein binding |
| Ssc.10552.1.A1_at | -1,14 | -0,86 | -1,28 | *PTPRG* | 1,02E-05 | 1,74E-04 | 2,97E-06 | Protein-tyrosine phosphatase gamma precursor | Hydrolase, protein phosphatase |
| Ssc.3139.1.A1_at | -1,13 | -0,91 | -0,67 | *RGS2* | 7,15E-04 | 3,73E-03 | 2,25E-02 | Regulator of G-protein signaling 2 | Signal transduction inhibitor |
| Ssc.26516.1.A1_at | 1,00 | 0,87 | 0,84 | *ABCG8* | 2,67E-04 | 8,86E-04 | 1,21E-03 | ATP-binding cassette, sub-family G, member 8 | Transport |
| Ssc.16332.1.S1_at | 1,01 | 0,71 | 0,90 | *ABCC2* | 2,40E-04 | 3,93E-03 | 6,43E-04 | Canalicular multispecific organic anion transporter 1 | Transport |
| Ssc.17339.1.S1_at | 1,01 | 0,81 | 0,98 | *SLC15A1* | 7,79E-05 | 5,73E-04 | 1,06E-04 | Oligopeptide transporter, small intestine isoform | Digestion, protein transport |
| Ssc.5656.1.S1_at | 1,14 | 0,87 | 0,73 | *TLL2* | 3,83E-04 | 3,36E-03 | 1,05E-02 | Tolloid-like 2 *[H. sapiens]* | Developmental protein, hydrolase, metalloprotease, protease |
| Ssc.11076.1.S1_at | -1,57 | -2,07 | -1,93 | *SDS* | 5,17E-03 | 6,43E-04 | 1,16E-03 | L-serine dehydratase | Gluconeogenesis, lyase |
| Ssc.2464.1.S1_at | -1,35 | -1,80 | -1,33 | *STC1* | 3,11E-03 | 3,07E-04 | 3,37E-03 | Stanniocalcin 1 precursor | Hormone activity, response to nutrient |
